# Leaves of Indoor Ornamentals Are Biodiversity and Functional Hotspots for Fungi

**DOI:** 10.3389/fmicb.2018.02343

**Published:** 2018-10-01

**Authors:** Alexander Mahnert, Rocel Amor Ortega, Christian Berg, Martin Grube, Gabriele Berg

**Affiliations:** ^1^Institute of Environmental Biotechnology, Graz University of Technology, Graz, Austria; ^2^Department of Biology, College of Science, University of the Philippines Baguio, Baguio, Philippines; ^3^Institute of Plant Sciences, Karl-Franzens-University, Graz, Austria

**Keywords:** indoor plants, phyllosphere mycobiome, antagonists, *Botrytis cinerea*, volatile organic compounds

## Abstract

Leaf-inhabiting fungi are an important, but often overlooked component of molecular biodiversity studies. To understand their diversity and function in relation to plant species and climate, the phyllospheres of 14 phylogenetically diverse ornamental plant species were analyzed under different controlled greenhouse conditions. We found unexpectedly high fungal diversity (H′ = 2.8–6.5), OTU numbers (449–1050) and abundances (10^3^–10^6^ CFU cm^-2^ leaf surface) associated with all plants studied indoors. Despite experimental limitations, the composition of fungal communities were inclined toward a plant species-dependent pattern compared to the ambient climatic variables. Most detected fungi were patho- and saprotrophs showing a yeast-like growth morphology and were associated to the groups of endophytes and potential plant pathogens in a plant species-specific manner. A representative strain collection showed that 1/3 of the tested fungi (mainly *Penicillium, Cladosporium*, and *Cryptococcus spp*.) were able to inhibit mycelial growth and 2/3 inhibit sporulation of the plant pathogen *Botrytis cinerea* by the production of antifungal volatile organic compounds (VOCs) completely. This study indicates that plant leaves harbor a stable phyllosphere fungal diversity in diverse microclimates and enrich distinctive functional guilds.

## Introduction

The phyllosphere represents one of the largest surface for microbial life on Earth (Lindow and Brandl, 2003) particularly leaves amount to a vast global surface area of around 10^9^ km^2^ (Woodward and Lomas, 2004). Despite being a hostile environment due to rapidly fluctuating environmental conditions such as solar radiation, temperature, humidity, and an overall limitation of nutrients, the phyllosphere supports diverse and complex microbial communities including many genera of bacteria, archaea, filamentous fungi, and yeasts (Lymperopoulou et al., 2016). Most studies describing phyllosphere microbial communities focus on bacterial colonizers of the leaf, though the leaf surface also harbors diverse fungal communities mainly studied by cultivation (Santamaría and Bayman, 2005). While foliar endophytic fungi are well-studied (Redman et al., 2002; Arnold, 2007; Crawford et al., 2010), no general conclusion has been formulated regarding major drivers of fungal composition in the phyllosphere of indoor environments. Studies on leaf-associated fungal communities of different tree species (oak, beech, poplar, apple, and pear) identified for instance land use (Jumpponen and Jones, 2009), host genetics (Cordier et al., 2012; U’Ren et al., 2012; Bálint et al., 2013; Vincent et al., 2016), tissue age (U’Ren and Arnold, 2016; Arrigoni et al., 2018) or host defense compounds (Saunders and Kohn, 2009) as important factors (Jumpponen and Jones, 2009; Cordier et al., 2012; Bálint et al., 2013). However, for the vast majority of leaf-associated fungi the ecological driver is unknown and, in general, plant-associated fungi are an often overlooked component of molecular biodiversity studies (Arnold and Lutzoni, 2007; Yahr et al., 2016).

Specifically, there is still limited information about the diversity of phyllosphere fungal communities on the surface of the leaves of indoor plants as well as the importance of leaf-associated fungi for the microbiome inside of built environments (Adams et al., 2013, 2014, 2015, 2017; Lymperopoulou et al., 2016). Recently we could show an impact of fungal abundance and diversity from plants on the microbiome of the built environment (Mahnert et al., 2015) and highlighted possible implications to the role of plants inside built environments and human health (Berg et al., 2014). However, among others, remaining uncertainties were in place for the stability of the microbiome in the phyllosphere in the presence of different microclimates and the proportion of suitable biocontrol agents on plant leaves for future biotechnological applications to control microbiomes indoors. To study the variability of the microbiome of indoor plants in relation to plant species and climate under different controlled conditions we selected different ornamental plant species grown in the greenhouses of the Botanical Garden in Graz (Austria). This study revealed the plant species as a potential driver for the composition of bacterial communities (Ortega et al., 2016). However, as reported for the rhizosphere (Berg et al., 2005), we expected a less pronounced plant species dependent effect for the mycobiome on plants in the built environment compared to Bacteria. Hence, our main initial hypotheses were (i) Fungal abundance and diversity would be less driven by the plant species compared to Bacteria; (ii) Fungal abundance and diversity would increase with warmer and wetter microclimates; and (iii) Fungi on leaves fulfill important functions regarding plant health.

Therefore, in this study we analyzed the fungal community composition on the leaf surface of 14 greenhouse plants grown in rooms with different controlled microclimates. This study design aims to show the effect of (A) room microclimate in a built system, and (B) effects of the plant host on the phyllosphere fungal community assembly. However, as each plant was only investigated in its naturally preferred microclimate, general validity of our insights are limited. We combined amplicon sequencing, cultivation and characterization of a strain collection to obtain a picture of fungal communities on 14 diverse plant species grown indoors. The study also focused on the functional characterization of antagonism against plant pathogens by volatile organic compounds (VOCs). They serve as ideal signaling molecules to facilitate both short- and long-distance intercellular and organismal interactions (Bitas et al., 2013) due to their ability to move through air spaces as well as liquids (Schmidt et al., 2015, 2016). *Botrytis cinerea* Pers. (1794) was selected as airborne target pathogens due to its broad host range and its enormous economic importance in the greenhouse as well as in the field (Dean et al., 2012).

## Materials and Methods

### Greenhouse Site Description and Plant Maintenance

Samples were collected from the greenhouse complex of the Botanical Garden of Graz at 47°04′55^′′^ N, 15°27′28^′′^ E, with an elevation of 378 m above sea level. More details can be taken from our previous study (Ortega et al., 2016). Briefly the greenhouse complex (**Supplementary Figure S1**) has four different houses simulating different terrestrial climatic conditions (**Supplementary Figures S2** and **S3**) and a nursery to grow young plants (sampled plant species per greenhouse can be seen in **Table 1**). Plant-care measures for the greenhouse plants included watering, application of fertilizers and a microbial pesticide. Two types of fertilizers were used to help maintain healthy plants: (1) is an NPK liquid fertilizer for foliar application (Wuxal^®^ Top N) and (2) is a water-soluble Phosphate and Potash nutrient [Hakaphos^®^ Rot 8 + 12 + 24 + (4)] applied in the soil. Application of these fertilizers also varied depending on the state of plant health. The biological bacterial-based pesticide DiPel^®^ was also used to protect the leaves of greenhouse plants from *Lepidoptera* larvae (caterpillar) that forages on them. A non-ionic surfactant (Break Thru^®^ S240) was applied to safeguard the effectiveness of the foliar fertilizer and microbial pesticide treatments. Both protocols were accomplished in all greenhouse areas except the Nursery.

**Table 1:** Plate counts (CFU/cm^-2^, averages with SD) of fungi from 14 different greenhouse plants.

Greenhouse room	Plant sample	CFU ± SD	
		SNA	Sab
Tropical	*Aechmea eurycorymbus*	^a^4.44E + 05 ± 7.00E + 04	^a^1.05E + 05 ± 1.47E + 04
	*Dracaena marginata*	^a^2.95E + 05 ± 4.20E + 04	^a^3.73E + 05 ± 9.51E + 04
	*Epipremnum aureum*	^a^1.23E + 05 ± 1.16E + 05	^a^6.63E + 04 ± 5.94E + 04
	*Musa paradisiaca*	^b^1.07E + 06 ± 1.59E + 05	^b^7.32E + 05 ± 8.51E + 04
Warm temperate	*Dracaena fragrans*	^a^1.45E + 04 ± 6.68E + 03	^a^2.26E + 04 ± 1.27E + 04
	*Howea forsteriana*	^a^3.36E + 04 ± 9.81E + 03	^a^3.58E + 04 ± 1.30E + 04
	*Malvaviscus penduliflorus*	^a^5.88E + 03 ± 3.40E + 03	^a^6.95E + 03 ± 1.94E + 03
Nursery	*Nephrolepis cordifolia*	^a^1.43E + 03 ± 1.17E + 03	^a^1.69E + 03 ± 1.37E + 03
Cold temperate	*Chlorophytum comosum*	^a^1.81E + 05 ± 7.76E + 04	^a^1.94E + 05 ± 8.28E + 04
	*Dracaena draco*	^a^2.27E + 04 ± 5.68E + 03	^a^3.96E + 04 ± 1.14E + 04
	*Olea europaea*	^a^2.37E + 04 ± 4.23E + 03	^a^3.84E + 04 ± 2.93E + 03
Succulents	*Aloe arborescens*	^a^2.81E + 04 ± 8.02E + 03	^a^2.86E + 04 ± 5.21E + 03
	*Beaucarnea recurvata*	^a^6.03E + 03 ± 1.18E + 03	^a^3.31E + 04 ± 1.58E + 04
	*Musa acuminata*	^a^9.84E + 02 ± 6.94E + 02	^a^4.50E + 02 ± 1.46E + 02

*Means that do not share the same letter (^*a*, *b*^) are significantly different (alpha = 0.05) according to a one-way ANOVA*.

14 greenhouse plants were selected due to their popular utilization as house plants. Leaves of these selected plants covered 8 different morphologies (fleshy, linear, oblong, lanceolate, cordate, orbicular, pinnate, and oval). While linear and oblong leaves were by far the most abundant morphotypes (five and threefold more frequent than the median, respectively). The sampled plants also varied in their respective ages (0 to 33 years, mean = 18 years, SD ± 11.7 years) and sizes (0.1 to 3 m^3^, mean = 1.6 m^3^, SD ± 0.9 m^3^).

### Sampling of Indoor Ornamentals

Leaves of 14 species of indoor plants were collected using sterile gloves and instruments. They were separated from the rest of the plant by cutting from the base of the petiole avoiding any possible contact with the leaf blade. Samples were placed inside 25 cm × 32 cm freezer bags (ARO Freezer Bags, Düsseldorf, Germany) immediately after collection and stored in a portable cooler with ice packs (Gio’Style Colombo Smart Plastics, Italy). All samples were immediately transported back to the laboratory for microbial isolation and DNA extraction.

Removal of microbial cells from leaves was conducted by placing 720 cm^2^ of a leaf inside a freezer bag (doubled as precaution from wear and tear) containing 50 ml 0.85% NaCl solution with Tween 80. Bags with leaves were then subjected to a series of steps including washing for 3 min (BagMixer Interscience, St. Nom, France) and sonication at 60 Hz for 3 min (Transsonic Digital T910 DH sonicator, Elma^TM^, Singen, Germany). The resulting microbial solution was then transferred to a 50 ml Sarstedt tube. For culture-dependent experiments, 100 μl of the solution was serially diluted ten-fold and plated on both Synthetic nutrient agar (SNA; 0.2 g Glucose, 0.2 g Sucrose, 1 g KH_2_PO_4_, 1 g KNO_3_, 0.5 g KCl, 0.5 g MgSO_4_ × 7 H_2_O, and 22 g agar per liter distilled water; adjusted to 5.5 pH with 1 M NaOH) and Sabouraud agar media (Carl Roth GmbH+Co. KG, Karlsruhe, Germany) in duplicates. The remaining microbial solution was then centrifuged (using Sorvall RC-5B Refrigerated Superspeed Centrifuge; DuPont Instruments^TM^, United States) at 6,169 *g* for 20 min and after transfer to 2.0 ml tubes at 18,000 *g* for 20 min to pellet cells. Pellets were stored at -70°C until DNA extraction.

### Internal Transcribed Spacer (ITS) Profiling

Genomic DNA was extracted using the FastDNA^®^ SPIN Kit for Soil (MP Biomedicals, Solon, OH, United States) as directed in the instruction manual. A total of 56 DNA samples were extracted; four replicates from different leaves of the same 14 plant species. PCR amplifications targeting the ITS region were conducted for each of the 56 samples using ITS1F (CTT GGT CAT TTA GAG GAA GTA A) and ITS2rP (GCT GCG TTC TTC ATC GAT GC) primers carrying sample-specific tags (Schoch et al., 2012). Using the thermocycler TC-Plus (TECHNE, Staffordshire OSA, United Kingdom), DNA was amplified in triplicate PCR reactions (30 μl each); 0.9 μl MgCl (25 mM), 6 μl Taq-&Go, 1.5 μl of 5 μM for each primer, 19.1 μl PCR water, and 1 μl of the DNA template with the following cycling conditions: 95°C, 5 min; 30 cycles of 95°C, 30 s; 58°C, 35 s; 72°C 40 s; and elongation at 72°C, 10 s). Amplicons from three independent reactions were then pooled and purified using the Wizard SV Gel and PCR Clean-Up System (Promega, Madison, WI, United States). PCR and DNA extraction controls were processed in parallel. However, as these controls did not result in any PCR products, control samples were not sequenced. Purified amplicon samples were further pooled in equimolar concentrations (520.8 ng DNA) and 50 μg of DNA in total was sent for Illumina MiSeq sequencing (Eurofins Genomics, Ebersberg, Germany) with chemistry version 2 (2 bp × 250 bp). Quality controls, indexing of PCR products, sequencing, library preparations and initial filtering of raw reads were conducted by Eurofins Genomics, Ebersberg, Germany (Ortega et al., 2016).

### Bioinformatics and Statistics

After initial quality control, raw reads were filtered, stitched and sorted according to respective barcodes. Raw reads are deposited as the project PRJEB19213 in the European Nucleotide Archive ^1^. Stitched reads were processed according to the fungal ITS analysis tutorial in QIIME 1.9.0 (Caporaso et al., 2010) as described before (Mahnert et al., 2015). After extracting barcodes per respective lengths, reads were demultiplexed, trimmed and filtered. Chimeric sequences were identified with usearch (Edgar, 2010) providing the QIIME formatted UNITE representative sequences (sh_refs_qiime_ver7_dynamic_01.08.2015) as a reference. Subsequently, all chimeric sequences were removed from the data set. Operational taxonomic units (OTUs) were picked against the UNITE reference given above using the blast algorithm and all remaining sequences were clustered *de novo*. The resulting OTU table was filtered for single and doubletons before it was rarefied to a depth of 10,419 sequences and served with all metadata as input for following alpha and beta diversity analysis and statistics. Adonis, ANOSIM (analysis of similarities), ANOVA (analysis of variances), PERMANOVA (permutational multivariate analysis of variance), MRPP (multi response permutation procedure), BioEnv, and mantel tests were calculated in QIIME and R (vegan package) with 999 permutations (Fierer et al., 2010; Oksanen, 2014; R Core Team, 2014). LEfSe analysis (Linear discriminant analysis effect size) was executed on a galaxy module provided by the Huttenhower Lab (Segata et al., 2011). Network analysis was realized in QIIME and visualized in Cytoscape 3.4.0 (Shannon et al., 2003). Ecological functions and guilds of fungi were annotated with FUNGuild (Nguyen et al., 2016).

### Cultivation and Isolation of Fungi

Serial dilutions of microbial solutions were plated on SNA and Sabouraud agar media, and incubated at room temperature for 5 days. Colony counts were expressed as CFU log10 cm^-2^ leaf. Colonies with a distinct phenotype were transferred to 2 ml Eppendorf tubes with 1 ml fungi-preservation solution [120 ml Glycerin, 40 ml 50% Glucose, 20 ml Peptone (20%), 20 ml Yeast extract (10%)]. Samples were numbered according to plant sample genus (except for *Aechmea eurycorymbus* where the common name Bromelia was used as a reference), origin, and isolation medium (e.g., Dth1SNA1: fungal isolate from *Dracaena* from the tropical house grown on SNA medium). All isolates were stored frozen at -70°C.

### Functional Characterization of Isolated Fungi

A two-clamp volatile organic compounds assay (TCVA) was performed as described before (Cernava et al., 2015) for analysis of the antagonistic volatiles produced by the isolated fungi. *Botrytis cinerea*, maintained on a Potato Dextrose Agar (PDA) was used as model pathogen for this study. Fungal inoculum was prepared by growing the fungus for 6 days on fresh PDA medium. After this period, the *B. cinerea* isolate was observed to have well-developed hyphae and was already sporulating.

A total of 629 fungal isolates were screened for their antagonistic activity against the pathogenic fungus *B. cinerea*. Isolates were transferred onto a new petri dish with PDA media and incubated for 6 days; to assure cleanliness of the isolates. A 5 mm sample from non-contaminated plates were cut and transferred onto a 6-well PDA plate and incubated at room temperature for 3-days. After the incubation period, plates with samples observed positive for growth were clamped together with newly made *B. cinerea* 6-well plates. *B. cinerea* containing 6-well plates were prepared by cutting 5 mm plugs from a 6-day old *B. cinerea* inoculum plate and placing it on the center of each well of a 6-well plate with Synthetic Nutrient-Poor Agar (SNA pH adjusted to 5.5). Set up of the plate-pair was carried out according to Cernava et al. (2015) in four replications. The set-up was incubated at room temperature for 3 days under dark conditions to eliminate any light-induced effect on the experiment. Inhibition of growth was then indicated as percentage (%).

### Characterization and Identification of Isolated Fungi

BOX-PCR fingerprint analysis of the antagonistic fungal isolates was implemented to avoid analysis of genetically similar strains. DNA was extracted by homogenization of fungal isolates using the FastPrep-24 Instrument (Illkirch, France) (30 s; 6 ms^-^). Homogenized samples were frozen (at -20°C for 30 min), heat-shocked at 100°C, and immediately centrifuged (16,000 *g* at 4°C for 5 min; HERMLE Labortechnik, Germany).

PCR amplification of 25 μl reaction mix (1 μl extracted DNA, 5 μl Taq-&Go, 2.50 μl 100 pmol/ml^-^ BOX A1R primer: 5′ CTA CGG CAA GGC GAC GCT GAC G 3′, and 16.50 μl PCR water) was realized using the TPersonal Combi, Biometra Thermocycler (Biometra GmbH, Germany) with an initial denaturation at 95°C for 6 min, followed by 35 cycles of 94°C, 1 min; 53°C, 1 min, and 65°C, 8 min with a final extension at 65°C for 16 min. The resulting BOX-PCR fingerprints were evaluated using the GelCompar II program (Kortrijk, Belgium). Cluster analysis was based on the unweighted pair-group average (UPGMA) algorithm.

### Gene Amplification and SANGER Sequencing

After DNA extraction, PCR amplification for each strain was performed using 30 μl of PCR reaction mix (0.9 μl MgCl (25 mM), 6 μl Taq-&Go, 1.5 μl of ITS1f primer (CTT GGT CAT TTA GAG GAA GTA A), 1.5 μl of ITS4r primer (TCC TCC GCT TAT TGA TAT GC), 19.1 μl PCR grade water, and 1 μl of the DNA template. Amplification was executed with an initial denaturation at 95°C for 5 min, followed by 30 cycles of 95°C for 30 s, 58°C for 35 s, and 72°C for 40 s with a final extension at 72°C for 10 min using a TC-Plus thermocycler (TECHNE, Staffordshire OSA, United Kingdom). Products were purified using the Wizard SV Gel and PCR Clean-Up System (Promega, Madison, WI, United States) and quantified on a Nanodrop 2000c spectrophotometer (PEQLAB, VWR International GmbH, Erlangen, Germany). Afterward, 14 μl of 40 ng μl^-1^ PCR product including one specific primer (ITS1f) was sent to LGC Genomics (Berlin, Germany) for sequencing.

Sequences were identified using the BLAST algorithm against the NCBI Targeted Loci Nucleotide BLAST – Internal transcribed spacer region (ITS) database^2^ and were deposited in GenBank at SUB2360857.

## Results

### Quantitative Aspects of Fungal Communities Associated With Indoor Plants

A high abundance of culturable fungi could be obtained from their phyllosphere. The highest abundance was found on the leaves of *Musa paradisiaca* with 1.07 × 10^6^ (SNA) CFU cm^-2^ and 7.32 × 10^5^ (Sabouraud) CFU cm^-2^ and the lowest on the leaves of *Musa acuminata* with 984 (SNA) and 450 CFU cm^-2^ (Sabouraud) as shown in **Table 1**. One-way ANOVA results (**Supplementary Table S1**) showed that there are significant differences in the fungal population densities on the phyllosphere of 14 greenhouse plants (*F crit = 2.5; F = 13.8; p-value < 0.05*). A Tukey’s HSD test also showed two groupings of population densities per plant (**Supplementary Table S2**) where *Musa paradisiaca* was grouped differently from the rest of the plants (**Table 1**).

A total of 6.01 × 10^6^ sequences were obtained from 56 phyllosphere samples by DNA-based amplicon sequencing after quality filtering. The average sequence per sample was 101,310; ranging from 3,967 to 250,429. In total, 14,220 OTUs could be defined, in a range of 590 to 4,162 and an average of 2,424 OTUs per sample (**Supplementary Table S3** for more details on read statistics). Both fungal isolates as well as sequences were assessed to study the composition and function of fungal communities associated with indoor ornamentals.

### The Composition of Fungal Communities Associated With Indoor Plants

**FIGURE 1:**
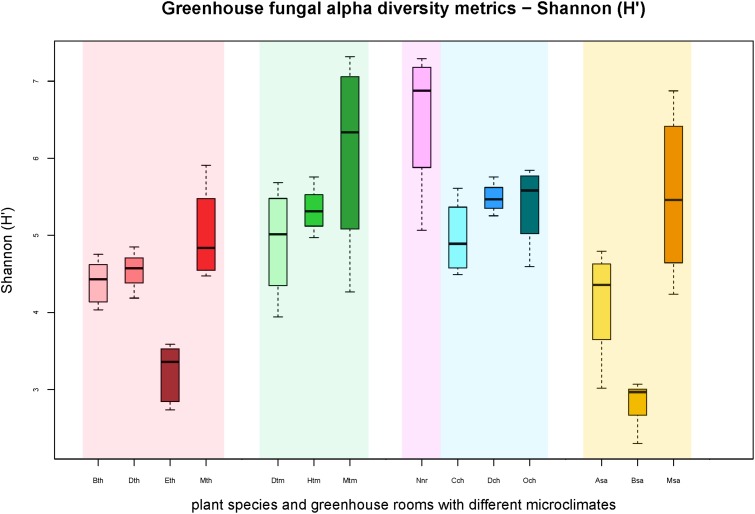
Boxplots of the Shannon diversity metric (H′) for individual plant species grouped according to respective greenhouse rooms (indicated by background colors: red – tropical house, green – warm temperate house, magenta – nursery, blue – cold temperate house, yellow – succulent house). Sample groups were abbreviated as: Bth, *Aechmea eurycorymbus*; Dth, *Dracaena marginata*; Eth, *Epipremnum aureum*; Mth, *Musa paradisiaca*; Dtm, *Dracaena fragrans*; Htm, *Howea forsteriana*; Mtm, *Malvaviscus penduliflorus*; Nnr, *Nephrolepis cordifolia* from a nursery room; Cch, *Chlorophytum comosum*; Dch, *Dracaena draco*; Och, *Olea europaea*; Asa, *Aloe arborescens*; Bsa, *Beaucarnea recurvata*; Msa, *Musa acuminata*.

All samples harbored a relatively high fungal alpha diversity. Computed Shannon indices of diversity (H’) varied from 4.2 in the succulent house up to 6.5 in the nursery room (**Figure 1**). Rarefaction analysis revealed that the number of observed OTUs covered 45.8–53.1% of the estimated taxonomic richness (Chao1; **Table 2**) as well as obvious variation in the fungal phyllosphere communities per greenhouse room and per plant species sample (**Supplementary Figures S4A–D**). Statistical analysis using a Two-sample T-Test showed that diversity (H’) of the fungal communities in the nursery room (6.5 H′) was significantly different from the cold temperate house (5.3 H′; *P* = 0.04) and the tropical house (4.4 H′; *P* = 0.01), but not to the other greenhouse rooms. On the other hand fungal diversity in the tropical house was significantly different from fungal diversity calculated for the warm temperate (5.4 H′; *P* = 0.04) and cold temperate houses (*P* = 0.02). No difference was determined between diversity estimates of the cold and warm temperate houses as well as the tropical and succulent houses (**Supplementary Tables S4–S6**). On the level of individual plant species, samples from the succulent and tropical houses showed higher variances than plant samples from the cold and warm temperate houses. However, no significant differences could be determined between all sampled plant species (**Supplementary Table S7**). Correlations of observed OTUs with the estimated taxonomic richness (Chao1) of each plant resulted in a coverage of 41.5–53%. Fungal diversity strongly varied per individual plant with *Nephrolepis cordifolia* showing the highest fungal diversity (H′ = 6.5) and *Beaucarnea recurvata* the lowest (H′ = 2.8) (**Table 3**).

**Table 2:** Alpha diversity of fungal communities in the phyllosphere of 14 greenhouse plants categorized per greenhouse room.

Greenhouse room	Seqs/Sample	Shannon index (H′)	Observed species (no. of OTUs)	Chao1 (no. of OTUs)	Coverage (%)
Cold temperate	10410	5.3	953.0	2056.4	46.3
Nursery		6.5	1000.8	1887.4	53.1
Succulents		4.2	652.6	1418.2	46.0
Warm temperate		5.4	918.2	1919.1	47.8
Tropical		4.4	646.1	1409.1	45.8

**Table 3:** Alpha diversity of fungal communities in the phyllosphere of greenhouse plants categorized per individual plant species.

Greenhouse room	Sample identifier	Origin	Seqs/Sample	Shannon index (H′)	Observed species (no. of OTUs)	Chao1 (no. of OTUs)	Coverage (%)
Tropical	Bth	*Aechmea eurycorymbus*	10410	4.4	615.5	1406.6	43.8
	Dth	*Dracaena marginata*		4.5	547.1	1163.9	47.0
	Eth	*Epipremnum aureum*		3.2	563.2	1358.7	41.5
	Mth	*Musa paradisiaca*		5.0	837.7	1694.6	49.4
Warm temperate	Dtm	*Dracaena fragrans*		4.9	900.4	1974.1	45.6
	Htm	*Howea forsteriana*		5.3	928.7	1979.3	46.9
	Mtm	*Malvaviscus penduliflorus*		6.1	925.6	1804.0	51.3
Nursery	Nnr	*Nephrolepis cordifolia*		6.5	1000.8	1887.4	53.0
Cold temperate	Cch	*Chlorophytum comosum*		5.0	791.9	1753.6	45.2
	Dch	*Dracaena draco*		5.5	1017.1	2190.8	46.4
	Och	*Olea europaea*		5.4	1050.0	2224.7	47.2
Succulents	Asa	*Aloe arborescens*		4.1	554.2	1315.6	42.1
	Bsa	*Beaucarnea recurvata*		2.8	448.5	1052.9	42.6
	Msa	*Musa acuminata*		5.5	955.0	1886.1	50.6

**FIGURE 2:**
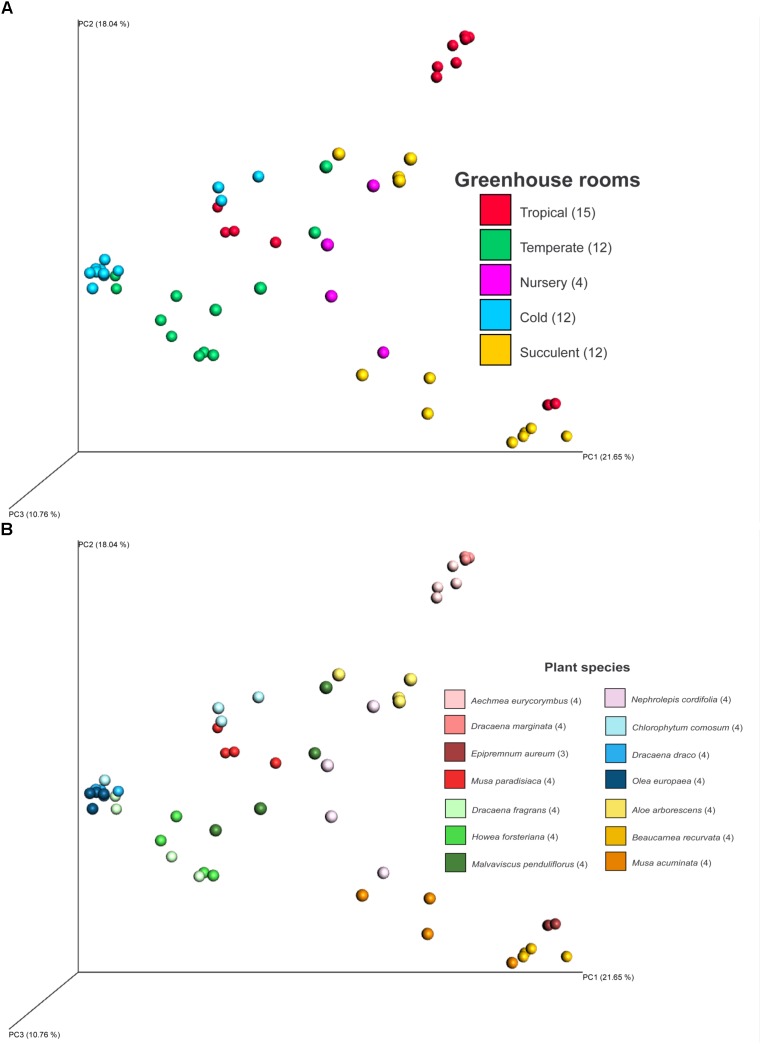
PCoA plot of fungal diversity in the phyllosphere of 14 greenhouse plants grown in different climatic conditions of a greenhouse complex. Shown samples are based on Bray-Curtis distances and colored according to different climates of different greenhouse rooms **(A)** and the sampled plant species **(B)**. PCoA axis are scaled according to explained diversity. Numbers in brackets refer to amount of samples after read normalization.

**FIGURE 3:**
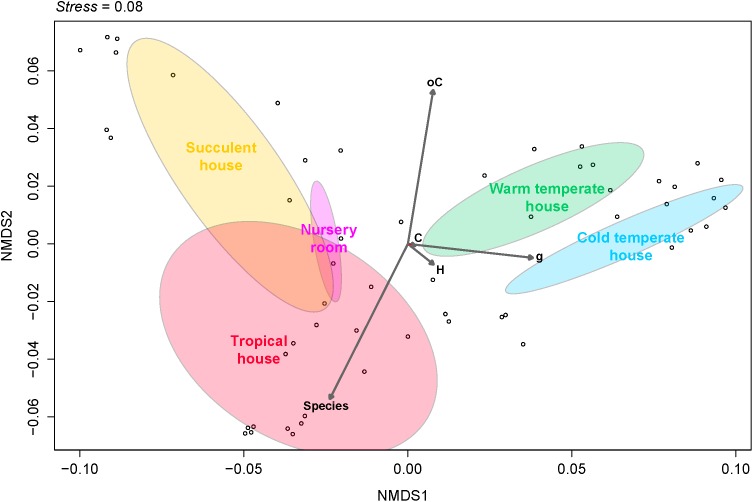
Non-metric multidimensional scaling (NMDS) plot derived from Bray-Curtis dissimilarities illustrating distances between fungal community compositions. The BIO-ENV vectors of environmental variables based on Euclidean distances represent the direction along the samples of each greenhouse room, showing the role each of them played in explaining the distribution of the samples and its directional influence. Species, plant species; H, relative humidity inside the room; C, temperature inside the room; oC, temperature outside the room; g, leaf weight.

Fungal beta diversity was examined to identify the drivers of fungal community composition. Ordination analysis based on Bray-Curtis dissimilarities showed inconspicuous clustering of the fungal communities per room or per plant species (**Figures 2A,B**). Phyllosphere fungal communities in the warm and cold temperate rooms were more similar to each other. Albeit relatively scattered, fungal communities from the tropical, nursery and succulent houses showed a reasonable similarity clustering as well. However, ANOSIM and PERMANOVA tests showed higher specificity of the phyllosphere fungal communities for different plant species (*P = 0.001, R = 0.89; pseudo-F = 12.4*) compared to different room microclimates (*P = 0.001, R = 0.40; pseudo-F = 6.3*). BIO-ENV analysis provided further evidence for the plant species as a potential driver of fungal community composition. In **Figure 3**, the vectors represent the Spearman rank correlations (ρs) of abiotic and biotic factors influencing the distribution of the fungal community on the leaf surface of the greenhouse plants. According to BIO-ENV analysis, plant species had strong influence on the fungal population dynamics in the phyllosphere of the greenhouse plants, being the variable that best explains the distribution of the relative abundance of the fungal community (*BEST = 0.9205*; **Supplementary Table S8**). Plant samples from the tropical house best summarized a potentially higher influence of plant species on the fungal community composition of the phyllosphere compared to the ambient climatic conditions. Despite constant climatic conditions, fungal community similarity was inclined toward individual plant species (**Figures 2A,B**). This species effect was also visible in **Figure 3** where the vector representing “Species” indicates the strongest directional influence.

**FIGURE 4:**
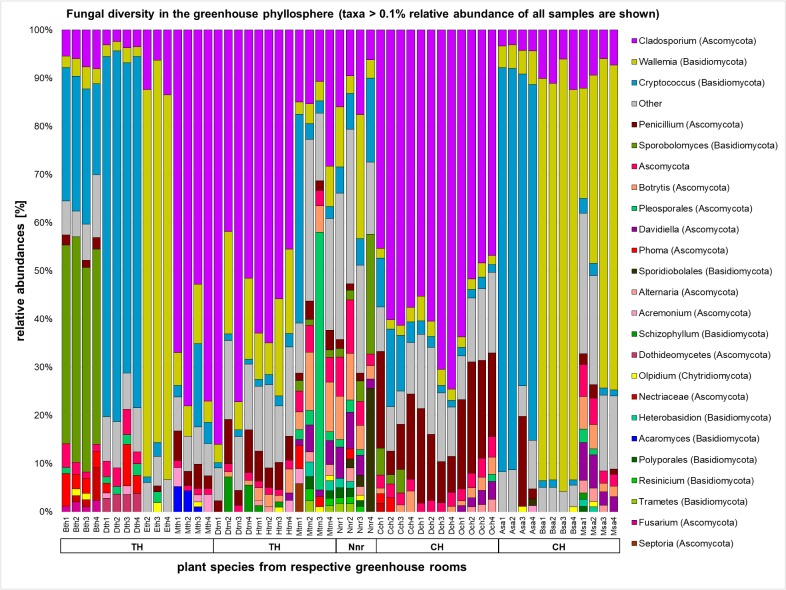
Fungal diversity in the phyllosphere of greenhouse plants represented by a bar chart. Highest resolved taxonomic assignments are shown for taxa with a higher relative abundance than 0.1% of all samples. Samples are sorted according to greenhouse rooms (TH, tropical house; TM, warm temperate house; Nnr, nursery; CH, cold temperate house; SA, succulent house), plant species (Bth, *Aechmea eurycorymbus*; Dth, *Dracaena marginata*; Eth, *Epipremnum aureum*; Mth, *Musa paradisiaca*; Dtm, *Dracaena fragrans*; Htm, *Howea forsteriana*; Mtm, *Malvaviscus penduliflorus*; Nnr, *Nephrolepis cordifolia* from the nursery room; Cch, *Chlorophytum comosum*; Dch, *Dracaena draco*; Och, *Olea europaea*; Asa, *Aloe arborescens*; Bsa, *Beaucarnea recurvata*; Msa, *Musa acuminata*) and respective replicates (1–4).

The majority of the fungal sequences were assigned to the order of *Capnodiales* (33.0%) from the phylum *Ascomycota*, and *Wallemialles* (20.14%) and *Tremellales* (16.71%) from the phylum *Basidiomycota*. Different rooms of the greenhouse complex showed distinct fungal profiles even on higher taxonomic levels (**Figure 4** and **Supplementary Figures S5, S6**). Whereas most plants of the greenhouse rooms were dominated on average by the ascomycete *Cladosporium* (12.31–57.95%) and the basidiomycete *Wallemia* (10.05–46.28%), divergent profiles were detected for plants in the tropical house (*Heterobasidion*; 27.99% and *Filobasidiales*; 23.12%), cold temperate house (*Penicillium*; 15.06%), and the succulent house (*Cryptococcus*; 26.50%). This observation found consonance on the level of individual plant species. Beside the overall prevalence of *Cladosporium* and *Wallemia, Aechmea eurycorymbus* revealed a high abundance of *Sporobolomyces* (42.76%) and *Cryptococcus* (25.63%). *Dracaena marginata* also grown in a tropical climate showed an exceptional high abundance of sequences assignable to the genus of *Cryptococcus* (72.27%). *Malvaviscus penduliflorus* from the warm temperate house exhibited high abundances of sequences from *Pleosporales* (15.11%). On the contrary, all three plants from the cold temperate house were characterized by the dominance of sequences from *Penicillium* [*Chlorophytum comosum* (13.02%), *Dracaena draco* (12.35%), and *Olea europaea* (19.83%)]. Distinct profiles of taxonomy could be also used to characterize 9 out of 14 plant species (*Aechmea eurycorymbus, Aloe arborescens, Beaucarnea recurvata, Dracaena fragrans, D. marginata, Malvaviscus penduliflorus, Musa paradisiaca, Nephrolepis cordifolia, and Olea europaea*) by fungal community features with a linear discriminant analysis of the effect size (LEfSe; **Supplementary Figure S7**). However, only three different greenhouse rooms could be discriminated by a much more downscaled set of fungal features.

### The Function of Fungal Communities Associated With Indoor Plants

#### Classification Into General Ecological Categories

**FIGURE 5:**
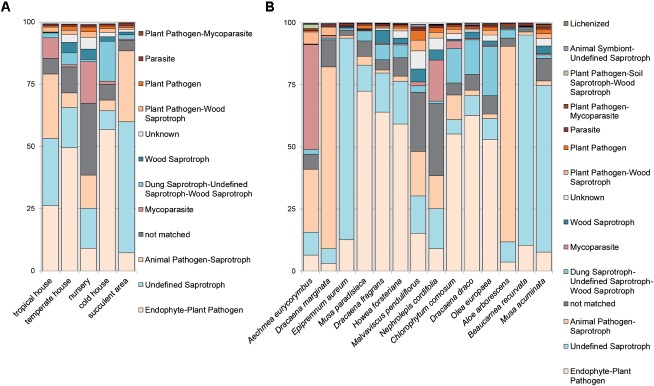
Analysis of fungal guilds with FUNGuild per greenhouse room **(A)** with different microclimates and per sampled plant species **(B)** for the category fungal guild. Fungal guilds were filtered to >1% relative abundance. X-axis shows samples per greenhouse room or plant species, respectively. Y-axis gives counts as relative abundances [%].

As revealed by the FUNGuild analysis (**Figures 5A,B** and **Supplementary Figures S8A–F**), most detected fungi were pathotrophs and saprotrophs showing a yeast-like growth morphology and were associated to the groups of endophyte-plant pathogens (*Musa paradisiaca, Dracaena fragrans, Howea forsteriana, Chlorophytum comosum, Dracaena draco*, and *Olea europaea*) and animal pathogen-saprotrophs (*Dracaena marginata* and *Aloe arborescens*). On the contrary, symbiotrophs were rare in the dataset (<1% relative abundance). Distribution patterns of the dominant ecologic guild functions were more similar between plants grown in the warm climate of the tropical and succulent house compared to temperate climates. Similarly, fungal traits could be brought into relation of the climatic conditions. Therefore many molds and soft rots were detected on plants from the cold temperate house (*Chlorophytum comosum, Dracaena draco*, and *Olea europaea*), white rots on plants from the warm temperate house (*Dracaena fragrans, Howea forsteriana*, and *Malvaviscus penduliflorus*) and soft rots on plants from the tropical climate (*Aechmea eurycorymbus* and *Dracaena marginata*). Samples of *Nephrolepis cordifolia* from the nursery room showed more unique compositions for many ecologic groups. Likewise, *Aechmea eurycorymbus* stood out with a relatively high abundance (42%) of mycoparasites.

#### Determination of the Antagonistic Potential Toward Pathogens

**FIGURE 6:**
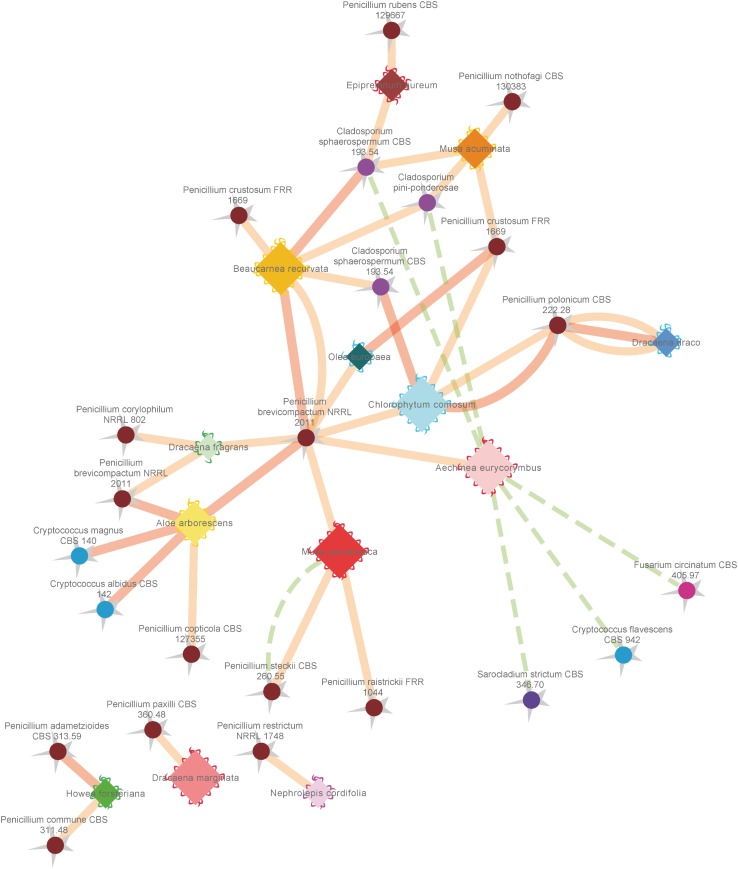
Network analysis of antagonistic and supportive effects of different fungal isolates from the phyllosphere of diverse greenhouse plants against the common plant pathogen *Botrytis cinerea*. Plant species are represented by diamonds with a waved line at their outer edges and respective fungal isolates are indicated by spheres with a zigzag line at the outside. Supportive (positive stimulating) effects on sporulation and growth are indicated by dashed green lines, while antagonistic (negative) effects on sporulation efficiency and growth of *Botrytis cinerea* are indicated by solid red (highest score) and orange (mean score) lines. Coloring of outer edges per plant species diamonds refers to respective greenhouse rooms with different microclimates. Fungal isolates were colored in warm (Actinomycota) and cold (Basidiomycota) colors. Edge width and node sizes were correlated with respective values for the intensity of effects (for edges) and number of isolates (for nodes). The network is based on the data shown in **Tables 4**, **5** and was visualized with an edge-weighted spring embedded layout in Cytoscape 3.4.0.

To investigate further functional aspects, we studied isolated fungal strains. A total of 629 fungal isolates from the phyllosphere of 14 greenhouse plants were screened for their antagonistic potential against *B. cinerea* using TCVA (Cernava et al., 2015). Observed effects included the inhibition of mycelial growth and sporulation (**Supplementary Figure S9**). **Table 4** summarizes the number of isolates per plant sample and the percentage of fungal strains that were tested positive for both antagonistic effects. Isolates from *Olea europaea* showed the highest percentage of antagonistic fungal strains where 33% of the total fungal isolates exhibited inhibitory effect on both mycelial growth and sporulation of *B. cinerea*. This study focused on identifying the 85 isolates that showed optimum antagonistic potential against the model pathogen. BOX-PCR fingerprinting and analysis, further divides these isolates into 39 genotypic groups at a cut-off level of 60%. These groups consist of three different classes including *Eurotiomycetes, Dothideomycetes*, and *Tremellomycetes*. Out of these genotypic groups, 17 species were identified by SANGER sequencing (**Table 5**). Frequently isolated antagonistic fungal species included: *Penicillium brevicompactum* (10), *Penicillium polonicum* (5), *Cladosporium sphaerospermum* (5), and *Penicillium crustosum* (4). It was also noted that *Penicillium* was highly represented with 13 different species namely *P. brevicompactum, P. paxilli, P. rubens, P. raistrickii, P. steckii, P. corylophilum, P. commune, P. adametzioides, P. restrictum, P. crustosum, P. polonicum, P. copticola*, and *P. nothofagi*. Three days after the initial set-up, the VOCs produced by these isolates decreased the fungal colony diameter by about 32.9–72.2% compared to the control; albeit ANOVA showed no significant differences between the inhibition percentages (**Supplementary Table S9**). During the initial screening for antagonistic potential, it was also observed that a few fungal strains promote rather than inhibit the growth of *B. cinerea*. After thorough investigation, six fungal strains all from the tropical house showed consistent growth promotion of *B. cinerea*; albeit very minimal and without significant differences (**Table 6**). BOX-PCR fingerprinting together with SANGER sequencing assigned these strains into six different species belonging to four different fungal classes. *Sarocladium strictum* and *Fusarium circinatum*; class *Sordariomycetes, Cladosporium sphaerospermum*, and *Cladosporium pini-ponderosae*; class *Dothideomycetes, Penicillium steckii*; class *Eurotiomycetes*, and *Cryptococcus flavescens*; class *Tremellomycetes*. All antagonistic and supportive interactions could be clearly visualized by a network analysis (**Figure 6**) based on the data shown in **Tables 5**, **6**.

**Table 4:** Percentages of fungal isolates with antagonistic properties against *Botrytis cinerea*.

Greenhouse room	Plant sample	Total isolates tested	Inhibits growth		Inhibits sporulation		Inhibits both growth and sporulation	
			No.	%	No.	%	No.	%
Tropical	*Aechmea eurycorymbus*	78	2	3	42	54	2	3
	*Dracaena marginata*	78	2	3	46	59	2	3
	*Epipremnum aureum*	29	3	10	21	72	2	7
	*Musa paradisiaca*	77	13	17	37	48	13	17
Warm temperate	*Dracaena fragrans*	16	4	25	9	56	4	25
	*Howea forsteriana*	24	6	25	14	58	5	21
	*Malvaviscus penduliflorus*	12	1	8	8	67	0	0
Nursery	*Nephrolepis cordifolia*	24	4	17	15	63	3	13
Cold temperate	*Chlorophytum comosum*	79	9	11	50	63	9	11
	*Dracaena draco*	21	5	24	13	62	5	24
	*Olea europaea*	18	6	33	12	67	6	33
Succulents	*Aloe arborescens*	53	16	30	23	43	15	28
	*Beaucarnea recurvata*	74	11	15	19	26	10	14
	*Musa acuminata*	46	10	22	12	26	9	20

**Table 5:** Taxonomic classification of fungal strains isolated from the phyllosphere of greenhouse plants with antagonistic effects against *Botrytis cinerea*.

Greenhouse room	Strains	Plant of origin	Score^a^	Species	Ident (%)	Accession
Tropical	Bth3Sab1	*Aechmea eurycorymbus*	++	*Penicillium brevicompactum* NRRL 2011	97	NR_121299.1
	Dth2Sab10	*Dracaena marginata*	++	*Penicillium paxilli* CBS 360.48	99	NR_111483.1
	Eth1Sab2	*Epipremnum aureum*	++	*Penicillium rubens* CBS 129667	99	NR_111815.1
	Eth4Sab3		++	*Cladosporium sphaerospermum* CBS 193.54	100	NR_111222.1
	Mth3Sab3	*Musa paradisiaca*	++	*Penicillium brevicompactum* NRRL 2011	97	NR_121299.1
	Mth4Sab3		++	*Penicillium raistrickii* FRR 1044	100	NR_119493.1
	Mth4Sab9		++	*Penicillium steckii* CBS 260.55	99	NR_111488.1
Warm temperate	Dtm3Sab6	*Dracaena fragrans*	++	*Penicillium brevicompactum* NRRL 2011	99	NR_121299.1
	Dtm4SNA9		++	*Penicillium brevicompactum* NRRL 2011	99	NR_121299.1
	Dtn3SNA1		++	*Penicillium corylophilum* NRRL 802	99	NR_121236.1
	Htm4Sab2	*Howea forsteriana*	++	*Penicillium commune* CBS 311.48	99	NR_111143.1
	Htm4SNA7		+++	*Penicillium adametzioides* CBS 313.59	100	NR_103660.1
Nursery	Nnr4SNA1	*Nephrolepis cordifolia*	++	*Penicillium restrictum* NRRL 1748	98	NR_121239.1
Cold temperate	Cch1Sab4	*Chlorophytum comosum*	++	*Penicillium crustosum* FRR 1669	99	NR_077153.1
	Cch1SNA2		++	*Penicillium polonicum* CBS 222.28	99	NR_103687.1
	Cch1SNA3		++	*Penicillium brevicompactum* NRRL 2011	99	NR_121299.1
	Cch3SNA8		+++	*Penicillium polonicum* CBS 222.28	100	NR_103687.1
	Cch4SNA8		+++	*Cladosporium sphaerospermum* CBS 193.54	100	NR_111222.1
	Dch3Sab5	*Dracaena draco*	+++	*Penicillium polonicum* CBS 222.28	100	NR_103687.1
	Dch3Sab8		++	*Penicillium polonicum* CBS 222.28	100	NR_103687.1
	Dch4Sab2		++	*Penicillium polonicum* CBS 222.28	99	NR_103687.1
	Och3SNA6	*Olea europaea*	++	*Penicillium brevicompactum* NRRL 2011	99	NR_121299.1
	Och4Sab4		+++	*Penicillium crustosum* FRR 1669	99	NR_077153.1
Succulents	Asa1SNA1	*Aloe arborescens*	+++	*Cryptococcus magnus* CBS 140	99	NR_130655.1
	Asa3SNA1		+++	*Penicillium brevicompactum* NRRL 2011	99	NR_121299.1
	Asa3SNA10		+++	*Penicillium brevicompactum* NRRL 2011	99	NR_121299.1
	Asa4Sab9		++	*Penicillium copticola* CBS 127355	99	NR_121516.1
	Asa4SNA6		+++	*Cryptococcus albidus* CBS 142	99	NR_111046.1
	Bsa2Sab3	*Beaucarnea recurvata*	+++	*Penicillium brevicompactum* NRRL 2011	97	NR_121299.1
	Bsa2Sab7		++	*Penicillium crustosum* FRR 1669	99	NR_077153.1
	Bsa2SNA4		+++	*Cladosporium sphaerospermum* CBS 193.54	99	NR_111222.1
	Bsa4Sab5		++	*Cladosporium pini-ponderosae*	97	NR_119730.1
	Bsa4SNA3		++	*Penicillium brevicompactum* NRRL 2011	99	NR_121299.1
	Bsa4SNA6		++	*Cladosporium sphaerospermum* CBS 193.54	100	NR_111222.1
	Msa2Sab7	*Musa acuminata*	++	*Penicillium nothofagi* CBS 130383	99	NR_121518.1
	Msa2SNA5		++	*Cladosporium sphaerospermum* CBS 193.54	100	NR_111222.1
	Msa3Sab6		++	*Cladosporium pini-ponderosae*	100	NR_119730.1
	Msa3SNA4		++	*Penicillium crustosum* FRR 1669	99	NR_077153.1

*^*a*^Inhibition score (++) indicates 31–60%; (+ + +) 61–80% mycelial growth inhibition on Botrytis cinerea*.

**Table 6:** Taxonomic classification of fungal strains isolated from the phyllosphere of greenhouse plants with positive effects on mycelial growth of *Botrytis cinerea*.

Greenhouse room	Strains	Plant of origin	Score^a^	Species	Ident (%)	Accession
Tropical	Bth2SNA6	*Aechmea eurycorymbus*	+	*Sarocladium strictum* CBS 346.70	99	NR_111145.1
	Bth2SNA7		+	*Cryptococcus flavescens* CBS 942	98	NR_130696.1
	Bth3Sab9		+	*Cladosporium sphaerospermum* CBS 193.54	100	NR_111222.1
	Bth3SNA2		+	*Fusarium circinatum* CBS 405.97	99	NR_120263.1
	Bth4SNA1		+	*Cladosporium pini-ponderosae*	99	NR_119730.1
	Mth1SNA4	*Musa paradisiaca*	+	*Penicillium steckii* CBS 260.55	99	NR_111488.1

*^*a*^Inhibition score (+) indicates 1–10% mycelial growth promotion on B. cinerea*.

**FIGURE 7:**
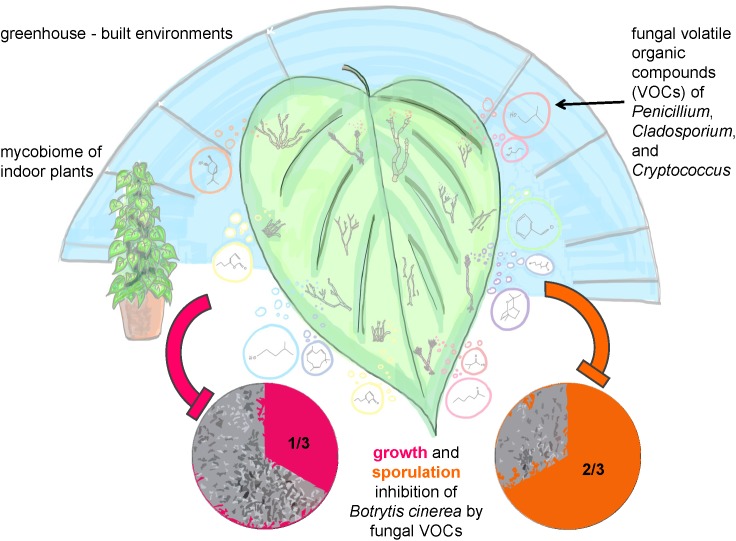
Sketch illustrating the study and main results of the structural and functional analysis of greenhouse plants in the built environment.

Conclusively, the main results and principles of our study were summarized in an illustrating sketch (**Figure 7**).

## Discussion

We detected high abundance and diversity of fungi in the phyllosphere of 14 phylogenetically different plant species grown under different climate conditions. The plant species was identified to have an influence on fungal community composition, and plant leaf morphology could be also positively correlated to similarities of the fungal community composition (**Supplementary Table S8**). Moreover, we showed that different plant species select for their fungal communities as well as for functional guilds in the phyllosphere.

Our data on the mycobiome of greenhouse ornamentals results in a falsification of two of our initial hypothesis. Therefore fungal communities were not only even more driven by the host species compared to Bacteria (Ortega et al., 2016), but fungal diversity also decreased in warmer and wetter microclimates against our initial assumptions. We suggest that the second phenomenon is dependent on the surrounding environment and climate. As the greenhouses were regularly window ventilated, most fungi could have been acquired from the outdoor environment. As the cold and warm temperate climates best reflect typical conditions of Austria and Central Europe, fungal diversity relatively increased toward the succulent and tropical houses with an exotic dry or warm and wet climate. Fungal communities inhabiting the phyllosphere of greenhouse plants varied not only across, but also within individual plants. Especially samples from *Nephrolepis cordifolia* showed relatively high variations of fungal community composition. This high variation may be attributed to the age of the leaves. Originating from the nursery room, *N. cordifolia* represents a plant species with relatively tight cycles of leaf renewal. This observation of high fungal variability within the leaf samples supports previous reports that younger leaves harbor a greater number of taxa than old leaves and could be explained by the release of nutrients from older leaves (Thompson et al., 1993). Hence, we suggest that the centered position of fungal samples from *N. cordifolia* in principal coordinate analysis plots might reflect a developing fungal community establishing itself on young plant leaves. However, plant age and plant size were not suitable to explain differences in the entire mycobiome. The strong influence of the plant species on the fungal community composition became more obvious by variations observed in the community composition within and across different greenhouse rooms. Fungal community composition differed more within than between rooms, implying that different species found inside the same room harbor individual fungal community compositions. This was further supported by results of the BIO-ENV analysis where the BEST value was highest for different plant species indicating that this factor best explains the distribution of fungal communities and additionally by the downscaled set of fungal discriminative features per greenhouse room according to the LEfSe analysis. However, worth to mention is the linkage of plant species and climate in our study. All plants were grown in their favored climate and not all the plant species were grown in all investigated microclimates except the genus *Dracaena*. Therefore, despite the significance of several applied statistical tests, the overall study design was not appropriate to discriminate between plant or microclimate specific drivers of the mycobiome. Furthermore, additional samples of the same plant species growing outdoors in close vicinity, non-leaf samples of indoor surfaces and indoor air, as well as a longitudinal analysis with additional metadata would have been necessary to identify the main driver behind fungal community assembly on indoor plant leaves and reveal potential dynamics of fungal composition in the phyllosphere. Nevertheless, our assumptions are supported by other studies (Dahlberg, 2001; Izuno et al., 2016). Moreover, correlation between plant leaf morphology and distances of fungal community further strengthens evidences of higher plant-host influence on the phyllosphere fungal community composition. *Dracaena fragrans, Dracaena draco* and *Howea forsteriana*, exhibiting the same linear, long, non-lobed, sword like (ensiform) leaf-shape and leaf size, harbored fungal communities that were more similar to one another. Similar correlation was observed in our previous study on bacterial communities in the phyllosphere of greenhouse plants (Ortega et al., 2016), however the correlation of the distances of fungal community to plant-leaf morphology was surprisingly even more stringent. These observation further supports previous reports that individual plants can have exclusive microbial associates possibly owing to their genetic make-up that ultimately controls their phenotypic characteristics and metabolism that is responsible for production of microbial attractants or defenses (Vorholt, 2012). These plant-driven fungal signatures were mainly assigned to the genera of *Cladosporium, Wallemia, Cryptococcus*, and *Penicillium* with saprotrophic and pathotrophic life styles associated to plants and animals. Distinct profiles were clearly evident for dominant fungi per plant species sample group and even for the ambient microclimate of the cold temperate house.

Our third hypothesis that fungi on leaves fulfill important functions regarding plant health could be confirmed and further developed. Our results showed that 1/3 of the tested fungi were able to inhibit mycelial growth and 2/3 inhibited sporulation of the plant pathogenic fungus *Botrytis cinerea* by VOCs production completely. Especially such high proportions of fungal isolates, which are capable to inhibit spore germination were never reported before and confirmed that the mycobiome is a crucial factor for plant health. Antifungal volatile production was especially present for fungal species belonging to the genera *Penicillium, Cryptococcus*, and *Cladosporium*. These species exhibited antagonistic effects to the pathogen *B. cinerea* by inhibiting its mycelial growth by approximately 30 to 80% with a visible reduction, if not total inhibition, of spore formation. Fungal genera of *Cryptococcus* (Buzzini et al., 2005) and *Cladosporium* (Paul and Park, 2013) were already shown to be potent VOC producers. In general, fungal VOCs were reported to have a brought set of functional features and characteristic odors ranging from antibiotic (6-Pentyl-α-pyrone, coconut odor), anti-microbial (Benzyl aldehyde, almond odor), or plant-growth promoting (β-Caryophyllene, woody-spicy odor) even to antifungal effects (1-Butanol-3-, methyl-, acetate, banana odor; Isobutyric acid, rancid cheese-like odor, 1,8-Cineole; camphor-like odor) (Morath et al., 2012). Especially 3- and 2-methyl-1-butanol compounds with antifungal effects were reported to be specific for different *Penicillium* sp. (Fischer et al., 1999). These extensive functional capabilities are useful for biological control strategies to defend plant pathogens or post-harvest fungal growth (mycofumigation). The biocontrol activity of *Penicillium and Cryptococcus* or of *Cladosporium* as a mycoparasite against *B. cinerea* has been already documented (Helbig, 2002; Rouissi et al., 2013). Remarkably, it was also observed that VOCs produced by *Cladosporium sphaerospermum, Cladosporium pini-ponderosae*, and *Penicillium steckii* have both negative and positive effects on the mycelial growth of *B. cinerea*. Apparently, fungal strains from the same lineage can have different bioactivity since they can differ significantly in their quantitative and qualitative secondary metabolite production (Dresch et al., 2015). Since fungal VOCs are produced during primary and secondary metabolism a strain-specific effect may have occurred causing the bipolar bioactivity against *B. cinerea* in this study. Nevertheless, additional investigation, like strain selection along with bioactivity testing and identification of VOCs, is recommended to further explain the opposing bioactivity of these three fungal species. In addition, the artificial setup to test fungal antagonism by VOCs against only one fungal pathogen in our study needs to be critically considered. Since fungi might produce a completely different blend of VOCs on plant leaves than on agar against various fungal pathogens (beyond *B. cinerea*, selected as a model due to its broad host range and its enormous economic importance), the validity of our results needs further investigations.

Results of our study have implications on several fields. They support the recent conclusion of Yahr et al. (2016) that leaf-associated fungi appear to be an important hotspot of biodiversity. Considering the plant species-specific component of fungal diversity and the existence of 400,000 plant species worldwide, we need much more knowledge about this hidden diversity. Moreover, a new report on plants around the world estimated that 21% of all plant species are likely threatened with extinction (Kathy and Willis Steve, 2016) and this development would obviously affect plant-specific fungi as well. The extraordinary high amount of VOC producers against *B. cinerea* that we could detect in our study supports this aspect and indicates the functional potential, which could get lost. While the importance of microbial diversity for plant health was already described (Poza-Carrion et al., 2013; Berg et al., 2017), the question of its importance for human health still needs to be elucidated. Nevertheless, the sheer size of the phyllosphere (a thousand fold of the terrestrial surface microbiome), implies a substantial contribution to ecosystem health.

## Availability of Data and Materials

The datasets generated and/or analyzed during the current study are available in the European Nucleotide Archive repository (See text footnote 1) under the project PRJEB19213 and in GenBank of NCBI (https://www.ncbi.nlm.nih.gov/genbank/) at SUB2360857.

## Author Contributions

AM contributed to samplings, data analysis, writing the manuscript. RO contributed to samplings and wet lab work. CB contributed to study design and as a botanical consultant. MG contributed as a mycological consultant. GB designed the idea and study and wrote the manuscript. All authors read and approved the final manuscript.

## Conflict of Interest Statement

The authors declare that the research was conducted in the absence of any commercial or financial relationships that could be construed as a potential conflict of interest.

## Footnotes

1 www.ebi.ac.uk

2 http://blast.ncbi.nlm.nih.gov/Blast.cgi
