# Supplementary material for: Leaves of Indoor Ornamentals Are Biodiversity and Functional Hotspots for Fungi
Source: Front Microbiol. 2018 Oct 1;9:2343. doi: 10.3389/fmicb.2018.02343 (PMC6174238; doi:10.3389/fmicb.2018.02343)
Supplement: Supplementary file 1 [file Data_Sheet_1.PDF]

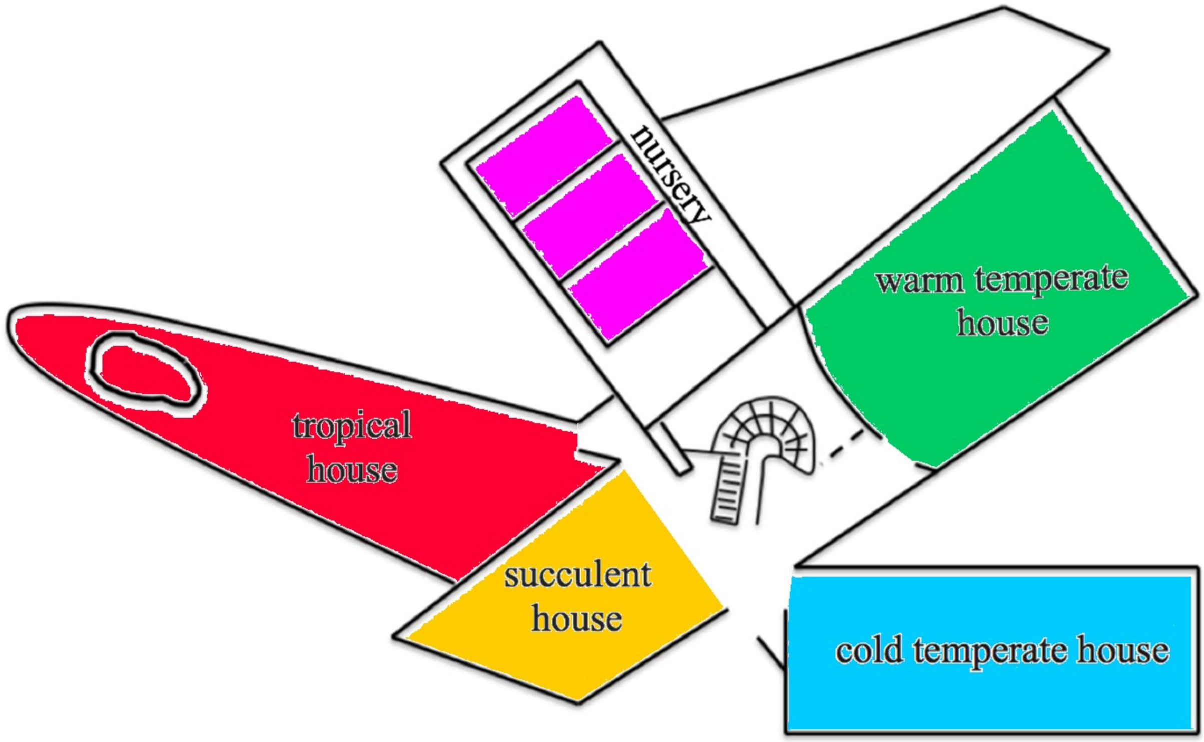

Additional file 1: Figure S1. The complex plan of the Botanical Garden of Graz greenhouse

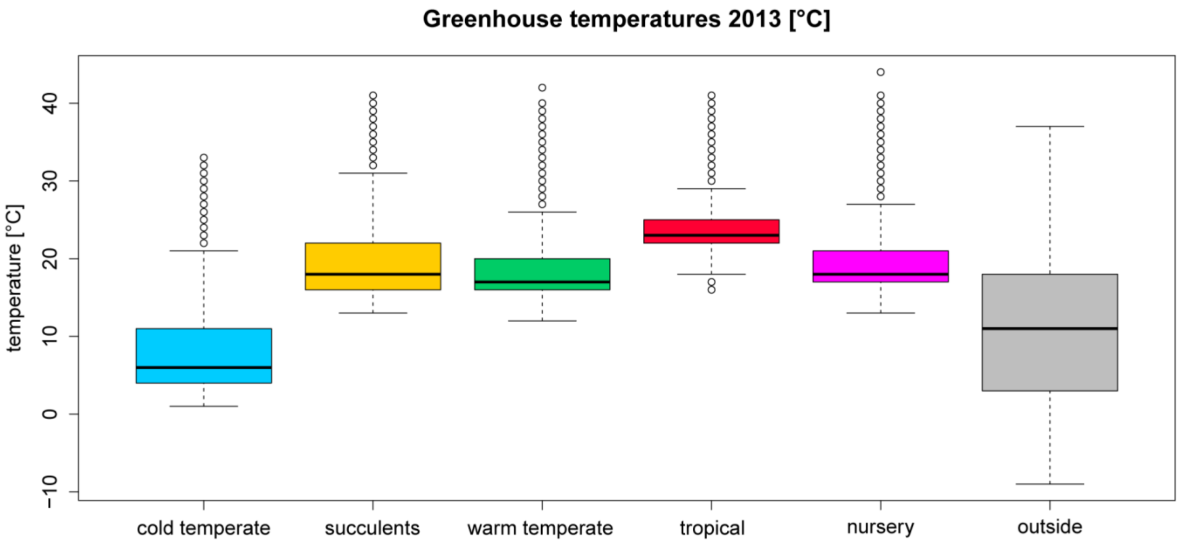

Additional file 2: Figure S2. Mean temperature inside each room of the greenhouse complex for the year 2013

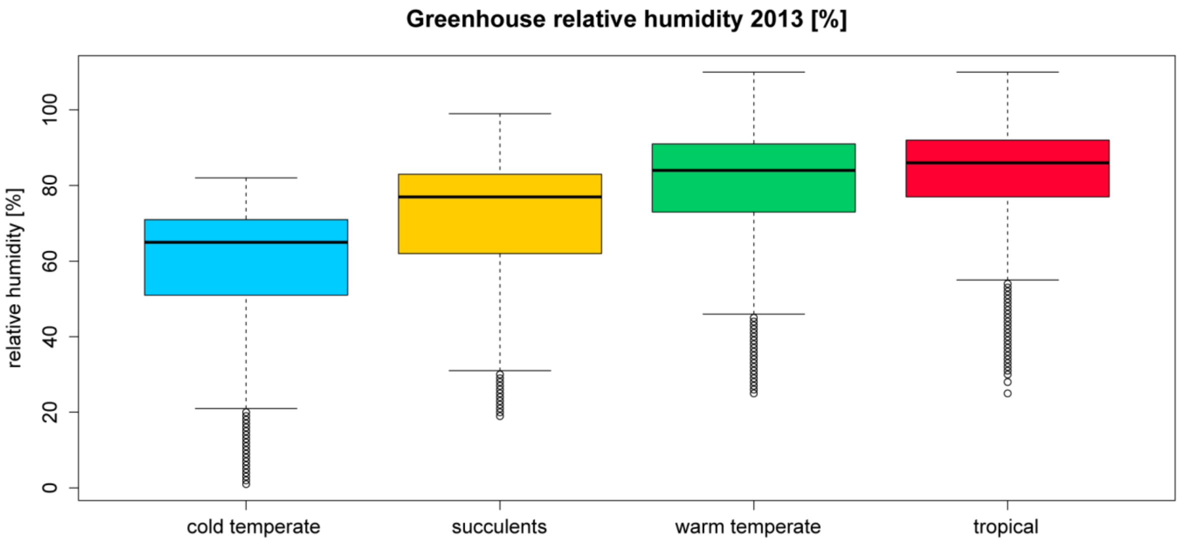

Additional file 3: Figure S3. Mean humidity inside each room of the greenhouse complex for the year 2013

Additional file 4: Table S 1. ANOVA of fungal CFU s from the phyllosphere of different greenhouse plants.

Source of Variation	SS	df	MS	F	p-level	F crit
Between Groups	1.5455E+12	13	1.18885E+11	13.84069	0.00001	2.50726
Within Groups	1.20253E+11	14	8.58951E+09			
Total	1.66575E+12	27				

Additional file 5: Table S2 . Tukey's test of fungal CFUs from the phyllosphere of different greenhouse plants

	Plant species	N	Subset for alpha 0.05	
			1	2
Tukey's HSD [a]	Msa	2	717.00	
	Nnr	2	1560.00	
	Mtm	2	6414.00	
	Dtm	2	18537.00	
	Bsa	2	19566.00	
	Asa	2	28368.00	
	Och	2	31050.00	
	Dch	2	31137.00	
	Htm	2	34677.00	
	Eth	2	94566.00	
	Cch	2	187765.50	
	Bth	2	274440.00	
	Dth	2	333900.00	
	Mth	2		901440.00
	Sig.		0.10	1.00

Means for groups in homogeneous subsets are displayed

a. Uses Harmonic Mean Sample Size = 2.0

Additional file 6: Table S3: Statistics on fungal sequences and observations (OTUs) of 56 samples from the phyllosphere of greenhouse plants.

Samples	Sequences	Samples	Observations (OTUs)
Eth1	3967	Eth1	590
Nnr1	10419	Nnr1	1073
Mtm4	19721	Eth3	1139
Bth3	25954	Bth3	1140
Eth3	29633	Mtm4	1428
Msa2	36534	Bsa3	1438
Nnr3	40847	Bth2	1534
Cch3	44772	Dth4	1629
Mth3	45470	Cch3	1690
Nnr2	48057	Asa3	1736
Htm3	50220	Dth3	1778
Mtm3	54825	Mtm3	1867
Bth2	55960	Msa2	1927
Och3	61333	Dth1	1936
Htm4	64387	Dth2	1961
Dtm3	68011	Bsa1	1988
Dtm2	69070	Bth4	2005
Asa3	72446	Nnr2	2076
Dch4	72564	Mth3	2093
Dch3	77070	Bth1	2136
Mtm2	83205	Nnr3	2182
Dth4	83553	Htm3	2231
Bth4	84209	Cch2	2241
Dth3	88308	Mth4	2288
Mth4	92971	Asa1	2341
Mtm1	93786	Htm4	2381
Msa1	95357	Bsa4	2392
Htm1	100582	Eth2	2412
Cch2	102038	Mtm1	2436
Dtm1	102635	Bsa2	2477
Cch1	108345	Dtm3	2478
Dth1	110702	Dtm1	2573
Bsa3	114802	Dch4	2616
Htm2	119601	Asa2	2677
Dch2	122489	Eth4	2697
Cch4	125845	Mtm2	2723
Mth1	128087	Cch1	2743
Dtm4	131339	Dtm2	2749
Och1	137822	Och3	2809
Bth1	139150	Asa4	2817
Bsa1	141646	Mth1	2878
Och2	148680	Msa4	2898
Och4	159577	Nnr4	2902
Msa4	160296	Dch3	2934
Dth2	161610	Htm1	3031
Eth2	161795	Msa1	3113
Dch1	161965	Htm2	3208
Nnr4	162144	Mth2	3274
Asa1	177489	Dtm4	3439
Asa4	185306	Och1	3612
Bsa4	187946	Msa3	3722
Bsa2	198314	Cch4	3775
Asa2	200691	Dch2	3920
Mth2	208753	Och4	3992
Eth4	227359	Och2	4119
Msa3	250429	Dch1	4162

Samples in total	Sequences in total	Samples in total	Observations (OTUs) in total
56	6010086	56	14220

Additional file 6: Table S3 Cont.:

Summary

	Sequences	Observations (OTUs)
Min	3967	590
Max	250429	4162
Median	101310	2424
Mean	107322.964	2471.536
Std. dev.	57704.355	795.872

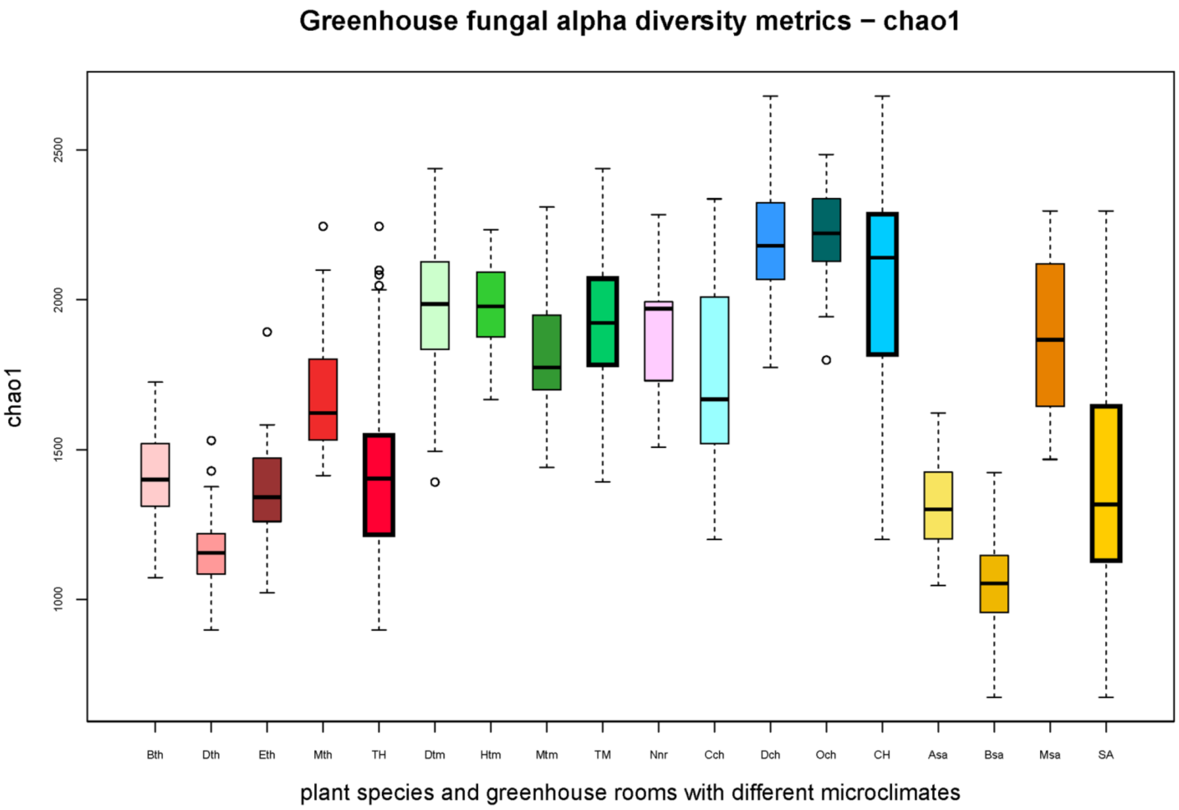

Additional file 7: Figure S4. Boxplots of A) estimated species (chao 1); B) observed species; C) the Simpson evenness; and D) the Simpson richness for individual plant species and different greenhouse rooms. Sample groups were abbreviated as: Bth: *Aechmea eurycorymbus*, Dth: *Dracaena marginata*, Eth: *Epipremnum aureum*, Mth: *Musa paradisiaca*, TH: tropical house, Dtm: *Dracaena fragrans*, Htm: *Howea forsteriana*, Mtm: *Malvaviscus penduliflorus*, TM: warm temperate house, Nnr: *Nephrolepis cordifolia* from a nursery room, Cch: *Chlorophytum comosum*, Dch: *Dracaena draco*, Och: *Olea europaea*, CH: cold temperate house, Asa: *Aloe arborescens*, Bsa: *Beaucarnea recurvata*, Msa: *Musa acuminata*, SA: succulent house.

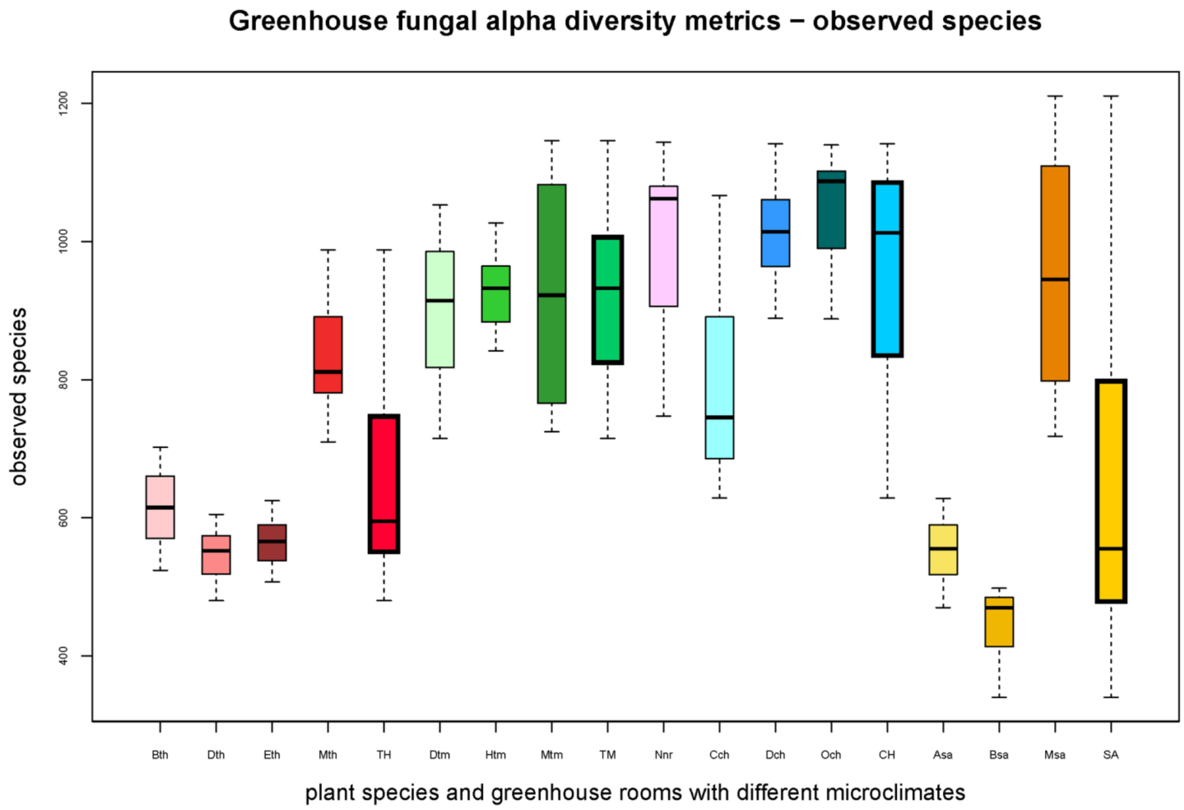

Additional file 7: Figure S4 B)

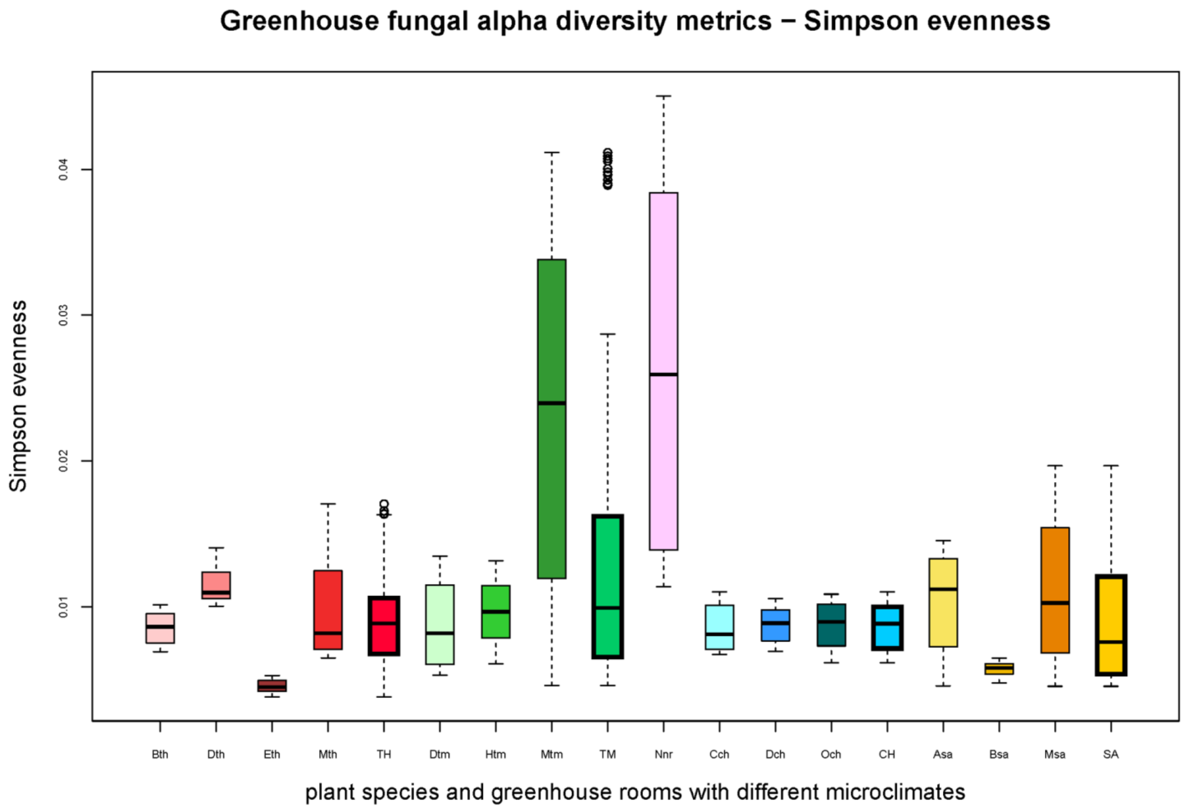

Additional file 5: Figure S4 C)

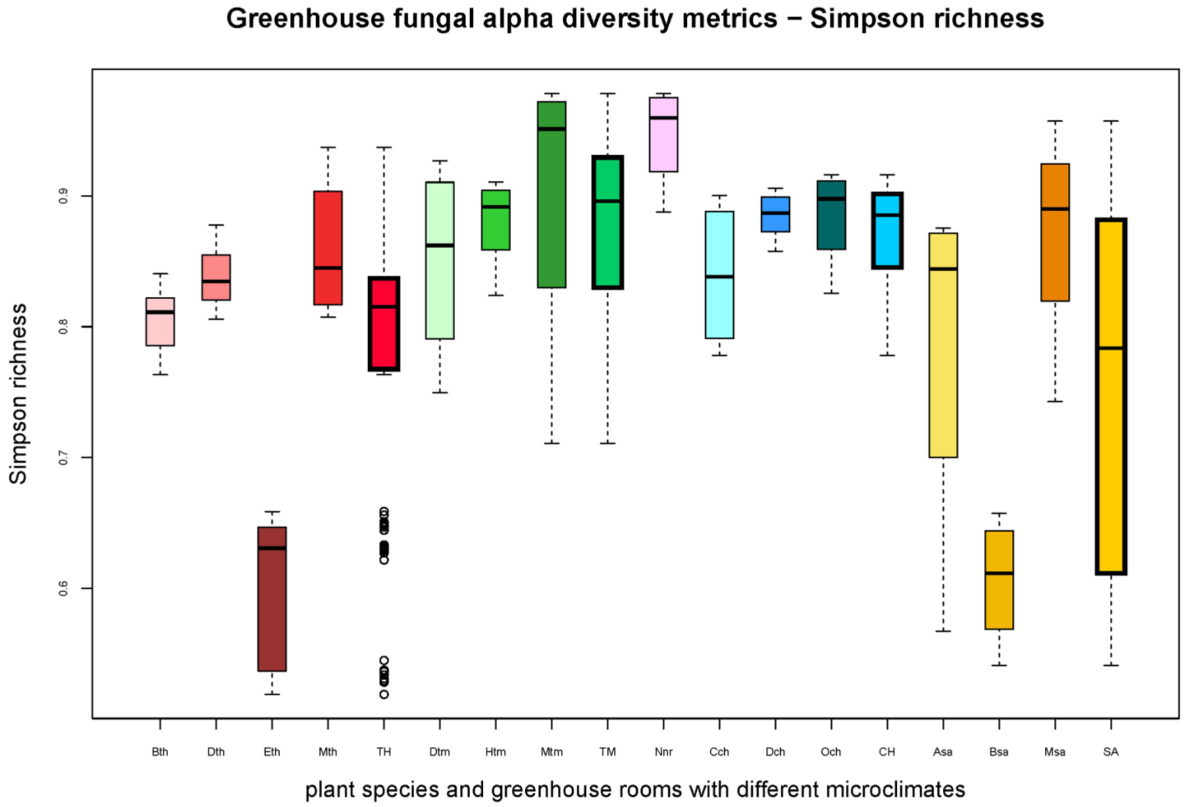

Additional file 7: Figure S4 D).

Additional file 8: Table S4. Statistical analysis of the Shannon index (H') per greenhouse room

Group1	Group2	Group1 mean	Group1 std	Group2 mean	Group2 std	t stat	p-value
Cold temperate	Tropical	5.29	0.43	4.37	0.71	3.82	0.02
Cold temperate	Warm temperate	5.29	0.43	5.44	0.90	-0.50	1.00
Cold temperate	Succulents	5.29	0.43	4.16	1.29	2.75	0.08
Cold temperate	Nursery	5.29	0.43	6.53	0.85	-3.55	0.04
Nursery	Warm temperate	6.53	0.85	5.44	0.90	1.98	0.73
Nursery	Tropical	6.53	0.85	4.37	0.71	4.91	0.01
Nursery	Succulents	6.53	0.85	4.16	1.29	3.20	0.07
Warm temperate	Succulents	5.44	0.90	4.16	1.29	2.70	0.13
Tropical	Succulents	4.37	0.71	4.16	1.29	0.52	1.00
Tropical	Warm temperate	4.37	0.71	5.44	0.90	-3.33	0.04

Additional file 9: Table S5. Statistical analysis of Observed Species per greenhouse room

Group1	Group2	Group1 mean	Group1 std	Group2 mean	Group2 std	t stat	p-value
Cold temperate	Tropical	952.98	153.10	646.05	129.87	5.42	0.01
Cold temperate	Warm temperate	952.98	153.10	918.24	114.36	0.60	1.00
Cold temperate	Succulents	952.98	153.10	652.56	242.09	3.48	0.02
Cold temperate	Nursery	952.98	153.10	1000.78	136.01	-0.52	1.00
Nursery	Warm temperate	1000.78	136.01	918.24	114.36	1.11	1.00
Nursery	Tropical	1000.78	136.01	646.05	129.87	4.55	0.01
Nursery	Succulents	1000.78	136.01	652.56	242.09	2.56	0.29
Warm temperate	Succulents	918.24	114.36	652.56	242.09	3.29	0.05
Tropical	Succulents	646.05	129.87	652.56	242.09	-0.09	1.00
Tropical	Warm temperate	646.05	129.87	918.24	114.36	-5.49	0.01

Additional file 10: Table S6. Statistical analysis of the Chao1 per greenhouse room

Group1	Group2	Group1 mean	Group1 std	Group2 mean	Group2 std	t stat	p-value
Cold temperate	Tropical	2056.37	288.44	1409.11	231.95	6.22	0.01
Cold temperate	Warm temperate	2056.37	288.44	1919.12	165.13	1.37	1.00
Cold temperate	Succulents	2056.37	288.44	1418.20	379.58	4.44	0.01
Cold temperate	Nursery	2056.37	288.44	1887.44	199.33	1.02	1.00
Nursery	Warm temperate	1887.44	199.33	1919.12	165.13	-0.29	1.00
Nursery	Tropical	1887.44	199.33	1409.11	231.95	3.57	0.05
Nursery	Succulents	1887.44	199.33	1418.20	379.58	2.21	0.48
Warm temperate	Succulents	1919.12	165.13	1418.20	379.58	4.01	0.02
Tropical	Succulents	1409.11	231.95	1418.20	379.58	-0.07	1.00
Tropical	Warm temperate	1409.11	231.95	1919.12	165.13	-6.18	0.01

Additional file 11: Table S7. Statistical analysis of the Shannon index per plant species

Group1	Group2	Group1 mean	Group1 std	Group2 mean	Group2 std	t stat	p-value
Asa	Dtm	4.141	0.635	4.920	0.631	-1.506	1.00
Asa	Cch	4.141	0.635	4.978	0.410	-1.919	1.00
Bsa	Dch	2.827	0.276	5.489	0.160	-14.432	1.00
Bsa	Cch	2.827	0.276	4.978	0.410	-7.536	1.00
Bsa	Asa	2.827	0.276	4.141	0.635	-3.284	1.00
Bsa	Mth	2.827	0.276	5.013	0.534	-6.290	1.00
Bsa	Dtm	2.827	0.276	4.920	0.631	-5.259	1.00
Bth	Dtm	4.394	0.256	4.920	0.631	-1.336	1.00
Bth	Dch	4.394	0.256	5.489	0.160	-6.278	1.00
Bth	Cch	4.394	0.256	4.978	0.410	-2.093	1.00
Bth	Asa	4.394	0.256	4.141	0.635	0.641	1.00
Bth	Mth	4.394	0.256	5.013	0.534	-1.808	1.00
Bth	Bsa	4.394	0.256	2.827	0.276	7.202	0.82
Cch	Dtm	4.978	0.410	4.920	0.631	0.135	1.00
Dch	Dtm	5.489	0.160	4.920	0.631	1.514	1.00
Dch	Asa	5.489	0.160	4.141	0.635	3.565	1.00
Dch	Cch	5.489	0.160	4.978	0.410	2.011	1.00
Dth	Mth	4.548	0.204	5.013	0.534	-1.407	1.00
Dth	Nnr	4.548	0.204	6.526	0.852	-3.911	1.00
Dth	Cch	4.548	0.204	4.978	0.410	-1.628	1.00
Dth	Bth	4.548	0.204	4.394	0.256	0.812	1.00
Dth	Bsa	4.548	0.204	2.827	0.276	8.673	1.00
Dth	Dch	4.548	0.204	5.489	0.160	-6.284	1.00
Dth	Asa	4.548	0.204	4.141	0.635	1.056	1.00
Dth	Dtm	4.548	0.204	4.920	0.631	-0.971	1.00
Dth	Och	4.548	0.204	5.400	0.454	-2.965	1.00
Eth	Dth	3.241	0.307	4.548	0.204	-5.705	1.00
Eth	Htm	3.241	0.307	5.333	0.253	-8.346	1.00
Eth	Mth	3.241	0.307	5.013	0.534	-4.343	1.00
Eth	Nnr	3.241	0.307	6.526	0.852	-5.386	1.00
Eth	Dtm	3.241	0.307	4.920	0.631	-3.587	1.00
Eth	Bsa	3.241	0.307	2.827	0.276	1.580	1.00
Eth	Cch	3.241	0.307	4.978	0.410	-5.204	1.00
Eth	Bth	3.241	0.307	4.394	0.256	-4.571	1.00
Eth	Dch	3.241	0.307	5.489	0.160	-10.599	1.00
Eth	Och	3.241	0.307	5.400	0.454	-6.004	1.00
Eth	Asa	3.241	0.307	4.141	0.635	-1.912	1.00
Eth	Mtm	3.241	0.307	6.066	1.139	-3.535	1.00
Htm	Bsa	5.333	0.253	2.827	0.276	11.588	1.00
Htm	Mth	5.333	0.253	5.013	0.534	0.939	1.00
Htm	Asa	5.333	0.253	4.141	0.635	3.021	1.00
Htm	Och	5.333	0.253	5.400	0.454	-0.224	1.00
Htm	Nnr	5.333	0.253	6.526	0.852	-2.325	1.00
Htm	Dch	5.333	0.253	5.489	0.160	-0.902	1.00
Htm	Cch	5.333	0.253	4.978	0.410	1.277	1.00
Htm	Dtm	5.333	0.253	4.920	0.631	1.053	1.00
Htm	Bth	5.333	0.253	4.394	0.256	4.520	1.00
Htm	Dth	5.333	0.253	4.548	0.204	4.187	1.00

Additional file 11: Table S7. Cont.

Group1	Group2	Group1 mean	Group1 std	Group2 mean	Group2 std	t stat	p-value
Msa	Bth	5.511	0.961	4.394	0.256	1.944	1.00
Msa	Dtm	5.511	0.961	4.920	0.631	0.890	1.00
Msa	Cch	5.511	0.961	4.978	0.410	0.883	1.00
Msa	Dth	5.511	0.961	4.548	0.204	1.697	1.00
Msa	Och	5.511	0.961	5.400	0.454	0.180	1.00
Msa	Mtm	5.511	0.961	6.066	1.139	-0.646	1.00
Msa	Nnr	5.511	0.961	6.526	0.852	-1.369	1.00
Msa	Mth	5.511	0.961	5.013	0.534	0.784	1.00
Msa	Asa	5.511	0.961	4.141	0.635	2.059	1.00
Msa	Htm	5.511	0.961	5.333	0.253	0.309	1.00
Msa	Eth	5.511	0.961	3.241	0.307	3.331	1.00
Msa	Bsa	5.511	0.961	2.827	0.276	4.647	1.00
Msa	Dch	5.511	0.961	5.489	0.160	0.039	1.00
Mth	Dtm	5.013	0.534	4.920	0.631	0.195	1.00
Mth	Cch	5.013	0.534	4.978	0.410	0.089	1.00
Mth	Dch	5.013	0.534	5.489	0.160	-1.478	1.00
Mth	Asa	5.013	0.534	4.141	0.635	1.819	1.00
Mtm	Bsa	6.066	1.139	2.827	0.276	4.786	1.00
Mtm	Asa	6.066	1.139	4.141	0.635	2.557	1.00
Mtm	Mth	6.066	1.139	5.013	0.534	1.450	1.00
Mtm	Htm	6.066	1.139	5.333	0.253	1.088	1.00
Mtm	Bth	6.066	1.139	4.394	0.256	2.481	1.00
Mtm	Cch	6.066	1.139	4.978	0.410	1.557	1.00
Mtm	Dch	6.066	1.139	5.489	0.160	0.869	1.00
Mtm	Dtm	6.066	1.139	4.920	0.631	1.525	1.00
Mtm	Och	6.066	1.139	5.400	0.454	0.941	1.00
Mtm	Nnr	6.066	1.139	6.526	0.852	-0.560	1.00
Mtm	Dth	6.066	1.139	4.548	0.204	2.273	1.00
Nnr	Dch	6.526	0.852	5.489	0.160	2.072	1.00
Nnr	Bsa	6.526	0.852	2.827	0.276	7.151	1.00
Nnr	Bth	6.526	0.852	4.394	0.256	4.150	1.00
Nnr	Asa	6.526	0.852	4.141	0.635	3.887	1.00
Nnr	Mth	6.526	0.852	5.013	0.534	2.606	1.00
Nnr	Och	6.526	0.852	5.400	0.454	2.019	1.00
Nnr	Dtm	6.526	0.852	4.920	0.631	2.624	1.00
Nnr	Cch	6.526	0.852	4.978	0.410	2.835	1.00
Och	Mth	5.400	0.454	5.013	0.534	0.957	1.00
Och	Bsa	5.400	0.454	2.827	0.276	8.380	1.00
Och	Dtm	5.400	0.454	4.920	0.631	1.071	1.00
Och	Dch	5.400	0.454	5.489	0.160	-0.319	1.00
Och	Bth	5.400	0.454	4.394	0.256	3.342	1.00
Och	Cch	5.400	0.454	4.978	0.410	1.195	1.00
Och	Asa	5.400	0.454	4.141	0.635	2.793	1.00

Additional file 12: Table S8: Combination of variables giving the largest Spearman rank correlations (ρs) of abiotic and biotic factors influencing the distribution of the fungal community on the leaf surface of 14 greenhouse plants (species = plant species; H = relative humidity inside the greenhouse room; g = leaf weight; oC = temperature outside the room; C = temperature inside the greenhouse room).

BEST variable combination (ρs)		
Variables	Size	Correlation
**species**	**1**	**0.9205**
species H	2	0.6759
species g oC	3	0.4878
species g C oC	4	0.3974
species g H C oC	5	0.3167

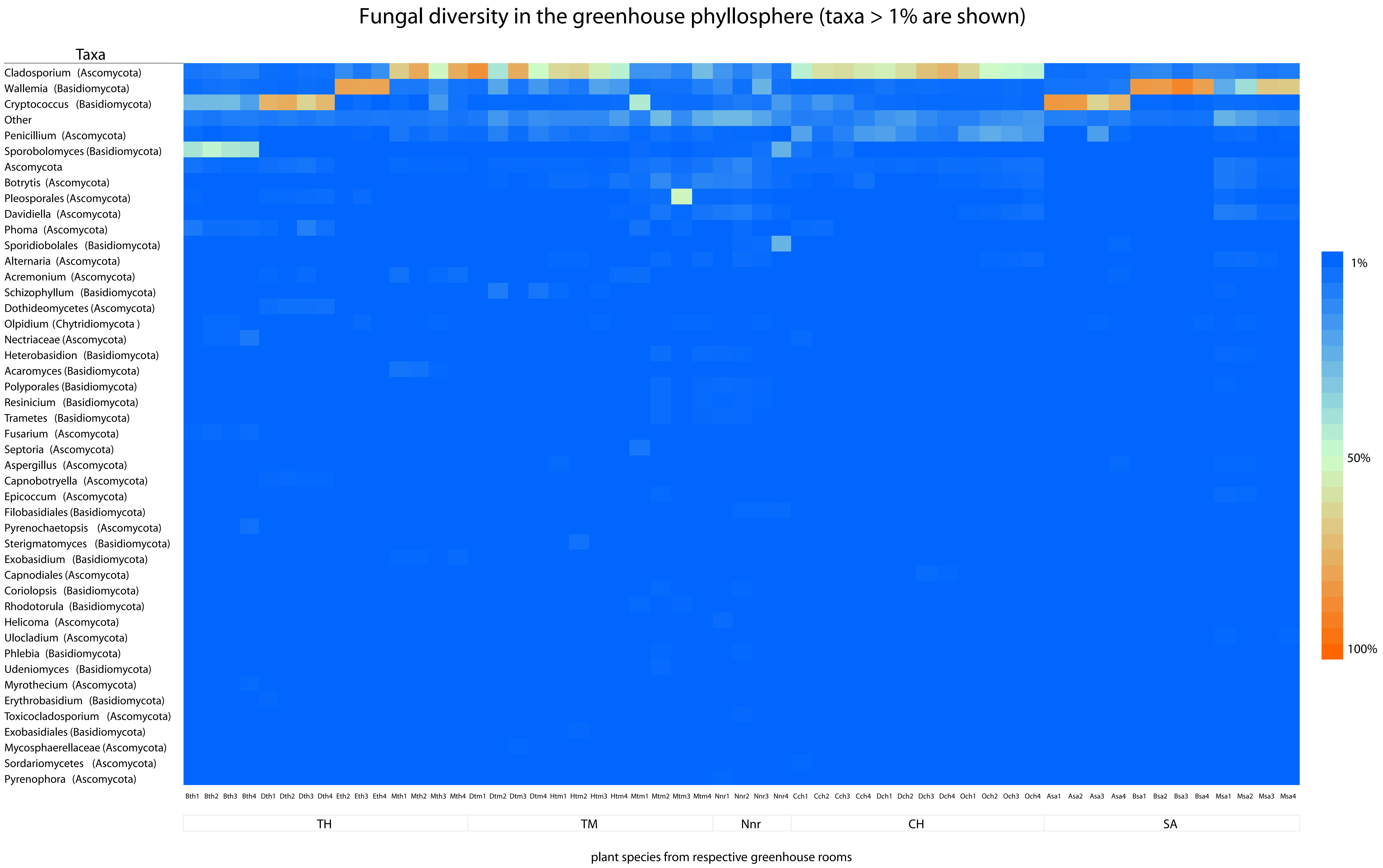

Additional file 13: Figure S5. Fungal diversity in the phyllosphere of greenhouse plants represented by a heatmap. Highest resolved taxonomic assignments are shown for taxa with a higher relative abundance than 1%. Samples are sorted according to greenhouse rooms (TH: tropical house, TM: warm temperate house, Nnr = Nursery, CH: cold temperate house, SA: succulent house), plant species (Bth: Aechmea eurycorymbus, Dth: Dracaena marginata, Eth: Epipremnum aureum, Mth: Musa paradisiaca, Dtm: Dracaena fragrans, Htm: Howea forsteriana, Mtm: Malvaviscus penduliflorus, Nnr: Nephrolepis cordifolia from the nursery room, Cch: Chlorophytum comosum, Dch: Dracaena draco, Och: Olea europaea, Asa: Aloe arborescens, Bsa: Beaucarnea recurvata, Msa: Musa acuminata) and respective replicates (1 – 4). Taxa are sorted according to respective relative abundances.

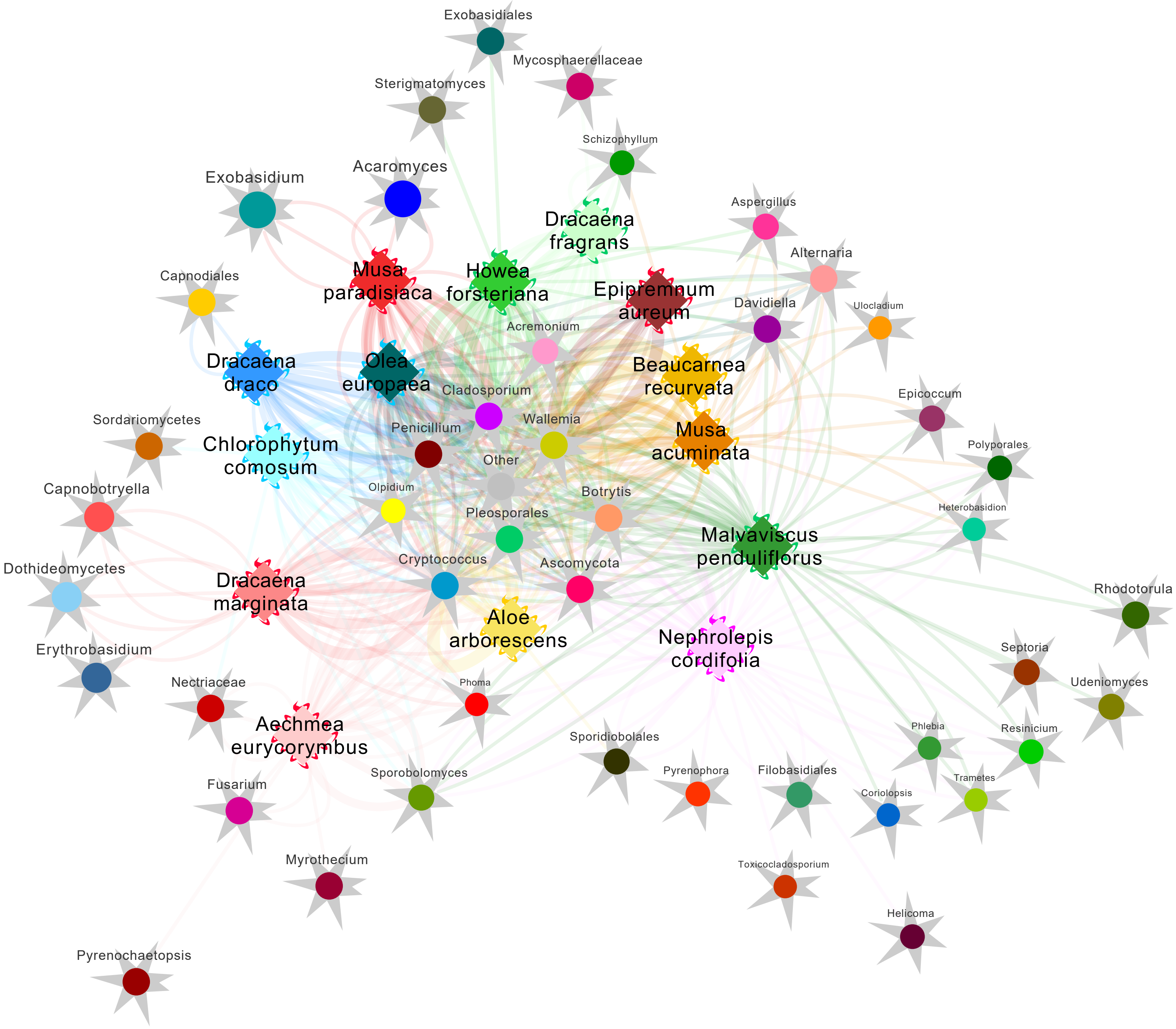

Additional file 14: Figure S6. Network analysis of fungal taxa with a relative abundance >1% from the phyllosphere of diverse greenhouse plants. Plant species are represented by diamonds with a waved line at their outer edges and fungal taxa are indicated by spheres with a zigzag line at the outside. Coloring of outer edges per plant species diamonds refers to respective greenhouse rooms with different microclimates. Fungal isolates were colored in warm (Actinomycota) and cold (Basidiomycota) colors. Edge width and transparency was correlated with respective eweights and node size corresponds to grown CFU per SNA plate. The network was visualized with an edge-weighted spring embedded layout in Cytoscape 3.4.0.

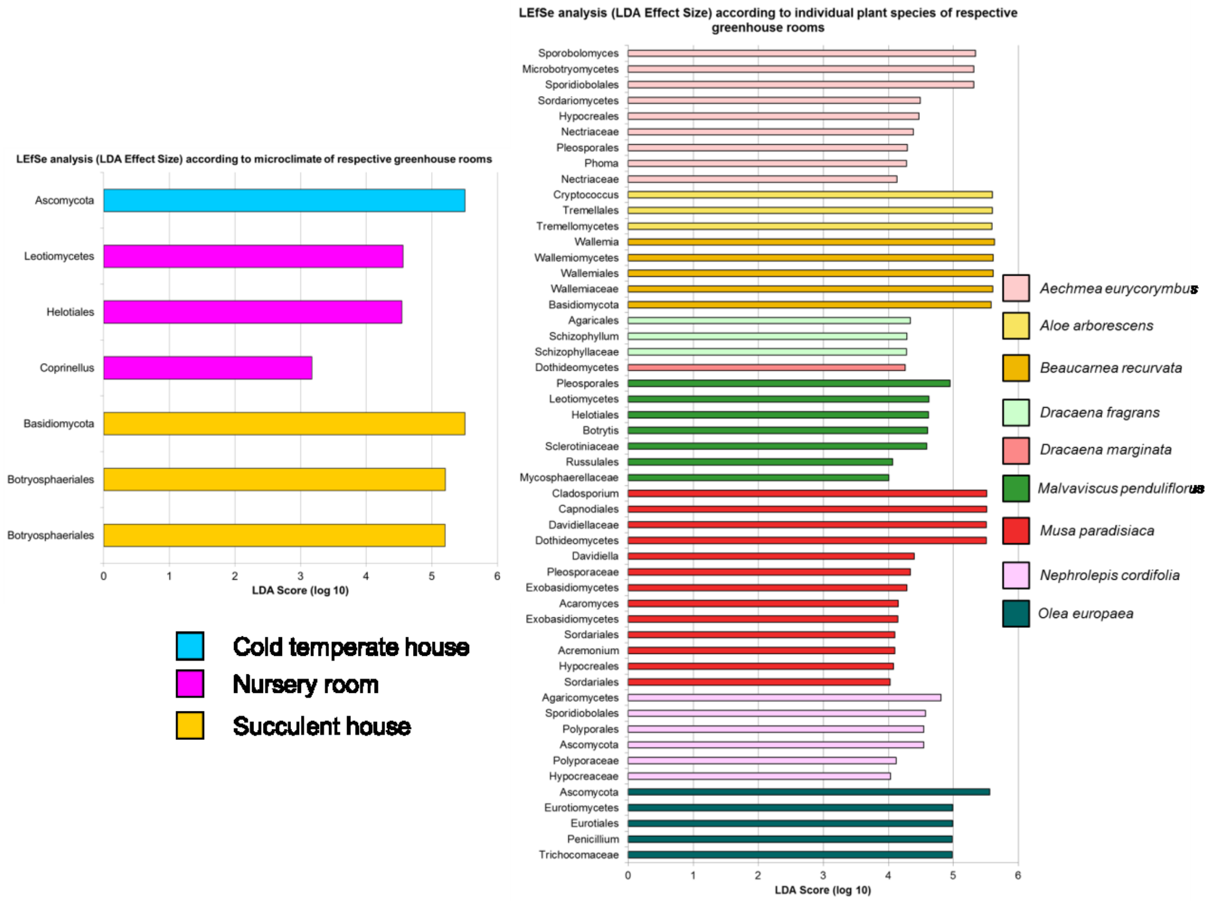

Additional file 15: Figure S7. Left: Linear discriminant analysis Effect Size (LEfSe) based on different microclimates of sampled greenhouse rooms (alpha value for the factorial Kruskal-Wallis test among classes: 0.05; alpha value for the pairwise Wilcoxon test between subclasses 0.05; threshold on the logarithmic LDA score for discriminative features: 2.0; strategy for multi-class analysis: all-against-all, more strict).

Right: Linear discriminant analysis Effect Size (LEfSe) based on sampled plant species in different greenhouse rooms (alpha value for the factorial Kruskal-Wallis test among classes: 0.05; alpha value for the pairwise Wilcoxon test between subclasses 0.05; threshold on the logarithmic LDA score for discriminative features: 4.0; strategy for multi-class analysis: all-against-all, more strict).

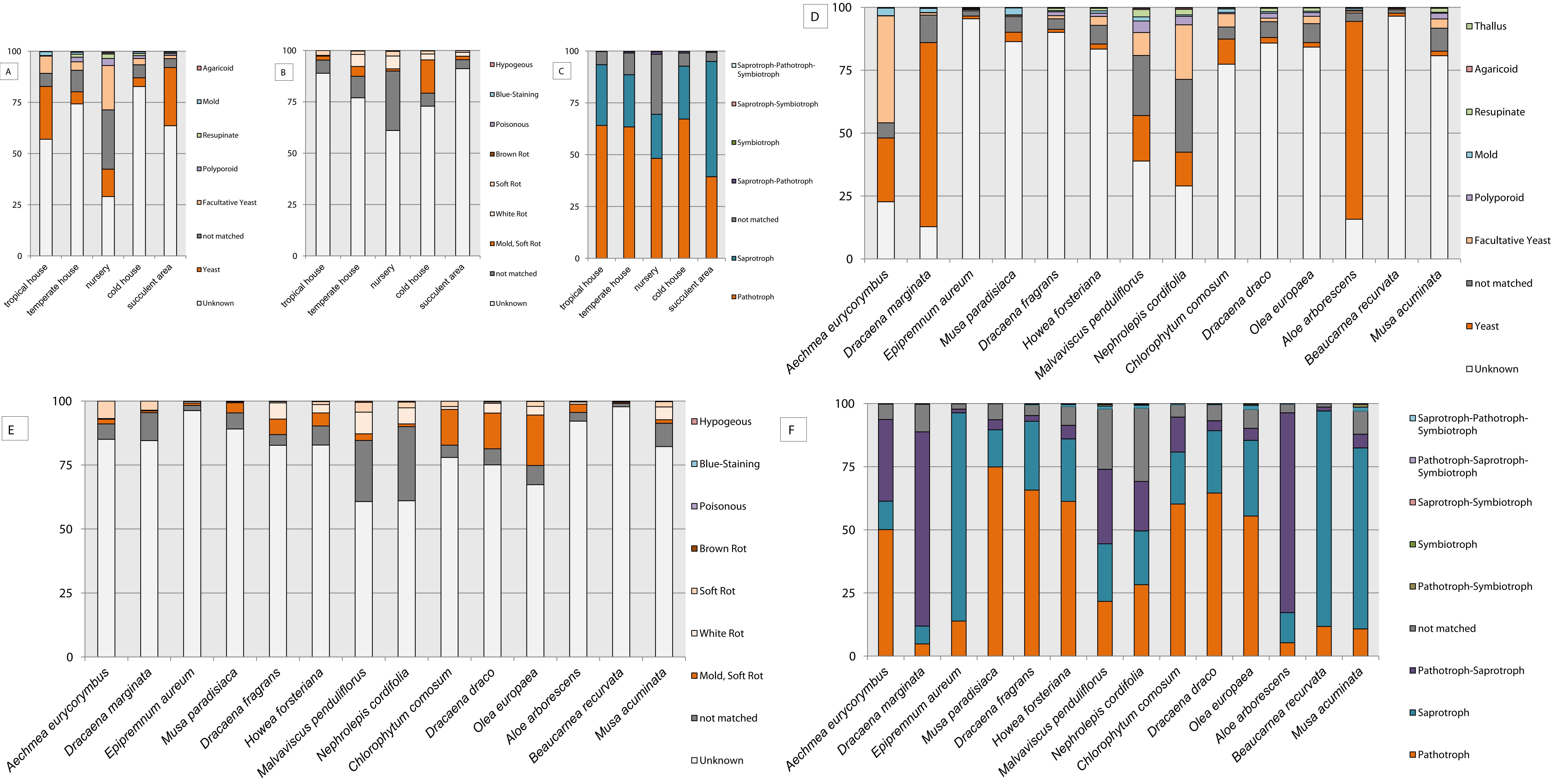

Additional File 16: Figure S8. Analysis of fungal guilds with FUNGuild per greenhouse room (A – C) with different microclimates and per sampled plant species (D - F) for the categories growth morphology (A and D), traits (B and E) and trophic mode (C and F). Category fungal growth morphology was filtered to >1% relative abundance.
X-axis shows samples per greenhouse room or plant species respectively. Y-axis gives counts as relative abundances [%].

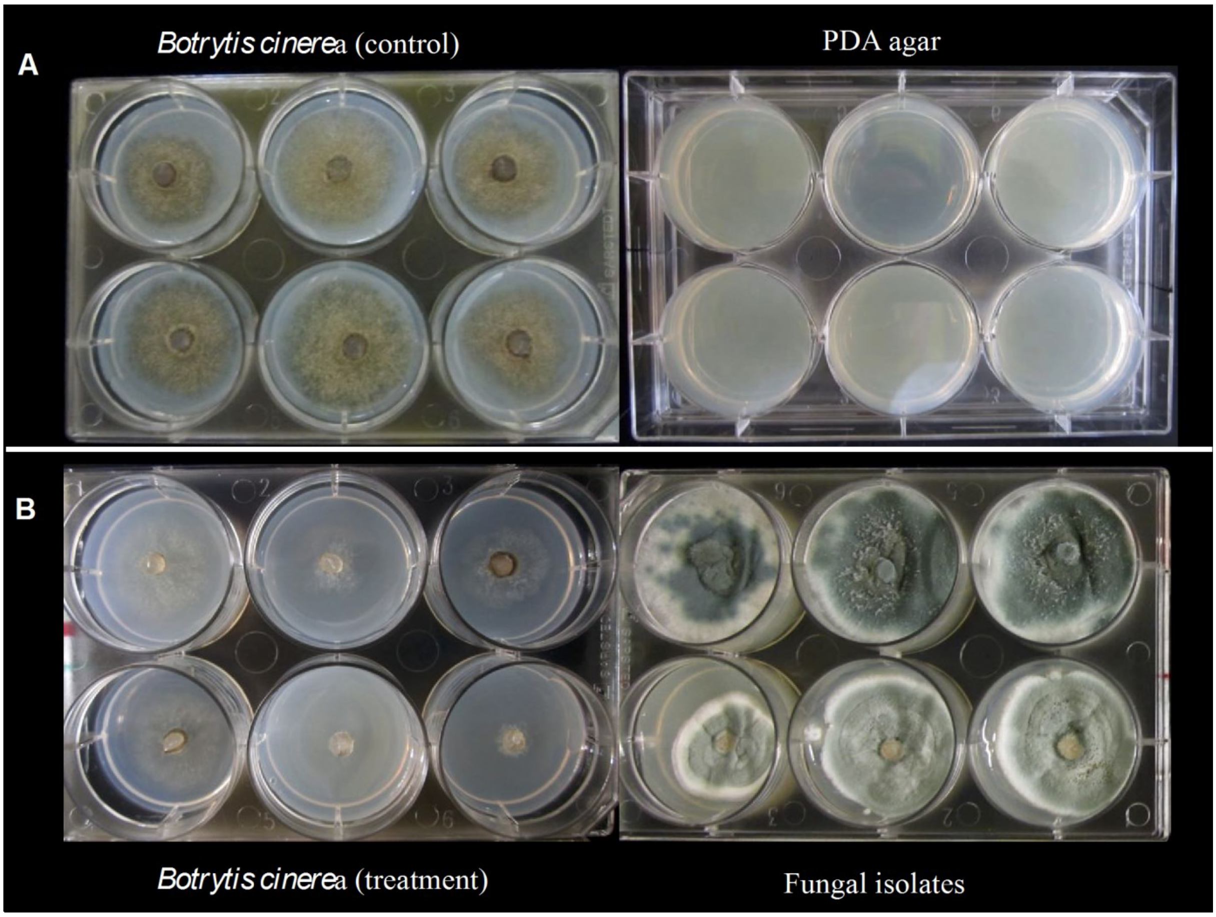

Additional file 17: Figure S9. Two-clamp VOCs assay of fungal isolates from greenhouse plants showing antifungal volatile activity against *Botrytis cinerea*. (A) *B. cinerea* plugs showing mycelial growth and spore germination (left), paired with an empty PDA plate (right).
(B) Volatile organic compounds produced by fungi (right) affected mycelial growth and germination of spores (left), compared to the control (above left).

Additional file 18: Table S9. ANOVA of the percent inhibition of antagonistic fungal strains against the growth of Botrytis cinerea

Source of Variation	SS	df	MS	F	p-level	F crit
Between Groups	15.6509	38	0.41187	1.35598	0.11074	1.50748
Within Groups	35.5375	117	0.30374			
Total	51.1884	155				
